# Patient reported outcomes for phosphomannomutase 2 congenital disorder of glycosylation (PMM2-CDG): listening to what matters for the patients and health professionals

**DOI:** 10.1186/s13023-022-02551-y

**Published:** 2022-10-29

**Authors:** C. Pascoal, I. Ferreira, C. Teixeira, E. Almeida, A. Slade, S. Brasil, R. Francisco, A. N. Ligezka, E. Morava, H. Plotkin, J. Jaeken, P. A. Videira, L. Barros, V. dos Reis Ferreira

**Affiliations:** 1 Portuguese Association for Congenital Disorders of Glycosylation (CDG), Lisbon, Portugal; 2 CDG & Allies—Professionals and Patient Associations International Network (CDG & Allies-PPAIN), Department of Life Sciences, NOVA School of Science and Technology, Universidade NOVA de Lisboa, 2829-516 Caparica, Portugal; 3 UCIBIO – Applied Molecular Biosciences Unit, Department of Life Sciences, NOVA School of Science andTechnology, Universidade NOVA de Lisboa, 2829-516 Caparica, Portugal; 4 Associate Laboratory i4HB - Institute for Health and Bioeconomy, NOVA School of Science and Technology, Universidade NOVA de Lisboa, 2829-516 Caparica, Portugal; 5 Student Support Office, Superior Institute of Engineering of Lisbon (ISEL), 1959-007 Lisbon, Portugal; 6 London, UK; 7 Department of Clinical Genomics, Mayo Clinic, Rochester, MN 55905 USA; 8 Glycomine, Inc, 733 Industrial Road, San Carlos, CA 94070 USA; 9 Center for Metabolic Diseases, Department of Pediatrics, KU Leuven, 3000 Leuven, Belgium; 10 CICPSI, Faculty of Psychology, University of Lisbon, Alameda da Universidade, 1649-013 Lisbon, Portugal

**Keywords:** Outcome assessment, Patient reported outcomes, Observer reported outcomes, Quality of life, Rare diseases, PMM2-CDG, People-centricity

## Abstract

**Background:**

Congenital disorders of glycosylation (CDG) are a growing group of rare genetic disorders. The most common CDG is phosphomannomutase 2 (PMM2)-CDG which often has a severe clinical presentation and life-limiting consequences. There are no approved therapies for this condition. Also, there are no validated disease-specific quality of life (QoL) scales to assess the heterogeneous clinical burden of PMM2-CDG which presents a challenge for the assessment of the disease severity and the impact of a certain treatment on the course of the disease.

**Aim and methods:**

This study aimed to identify the most impactful clinical signs and symptoms of PMM2-CDG, and specific patient and observer reported outcome measures (PROMs and ObsROMs, respectively) that can adequately measure such impact on patients’ QoL. The most burdensome signs and symptoms were identified through input from the CDG community using a survey targeting PMM2-CDG families and experts, followed by family interviews to understand the real burden of these symptoms in daily life. The list of signs and symptoms was then verified and refined by patient representatives and medical experts in the field. Finally, a literature search for PROMs and ObsROMs used in other rare or common diseases with similar signs and symptoms to those of PMM2-CDG was performed.

**Results:**

Twenty-four signs/symptoms were identified as the most impactful throughout PMM2-CDG patients’ lifetime. We found 239 articles that included tools to measure those community-selected PMM2-CDG symptoms. Among them, we identified 80 QoL scales that address those signs and symptoms and, subsequently, their psychometric quality was analysed. These scales could be applied directly to the PMM2-CDG population or adapted to create the first PMM2-CDG-specific QoL questionnaire.

**Conclusion:**

Identifying the impactful clinical manifestations of PMM2-CDG, along with the collection of PROMs/ObsROMs assessing QoL using a creative and community-centric methodology are the first step towards the development of a new, tailored, and specific PMM2-CDG QoL questionnaire. These findings can be used to fill a gap in PMM2-CDG clinical development. Importantly, this methodology is transferable to other CDG and rare diseases with multiple signs and symptoms.

**Supplementary Information:**

The online version contains supplementary material available at 10.1186/s13023-022-02551-y.

## Background

The World Health Organization defines Quality of Life (QoL) as “an individual’s perception of their position in life in the context of the culture and value systems in which they live and in relation to their goals, expectations, standards and concerns” [1]. One of the aspects of QoL is health-related quality of life (HrQoL). HrQoL is a multi-domain concept that encompasses physical, emotional, mental, and social functioning. It can be measured in a variety of ways, such as general scales, disease- or symptom-specific tools, which reflect upon the subjective perspective of a person regarding their condition [2]. Although general scales can be used for different diseases, they are less sensitive to detect small, yet important clinical differences in treatment effects [3]. These important differences are better measured using disease- or symptom-specific HrQoL scales, which will be more sensitive as they assess specific hallmarks of the disease or symptom. Concerning rare diseases, the study of QoL is challenging due to methodological issues as well as to limited literature on those conditions. Small patient populations, disease heterogeneity and scarcity of medical knowledge and specialists hamper the understanding of the burden of these diseases [4, 5]. This highlights the importance of ensuring a community-centric approach, including the professionals’ experience and the patients’ voice. Involving both stakeholder groups not only maximizes data collection but also data meaningfulness, ultimately contributing to the creation of sensitive and disease-tailored QoL tools. This is vital to delivering and appraising potential therapeutics.

Patient-reported outcome measures (PROMs) and observer-reported outcome measures (ObsROMs) are quantitative tools to obtain reports of patient outcomes directly from patients or their family/professional caregivers, respectively. They have been increasingly utilised as clinical endpoints, particularly with the aim to detect changes in the HrQoL in response to treatments [6]. They allow a deeper understanding of treatment impact and report domains that are not just clinically important but also meaningful for the patients [7]. They have been extremely useful, especially in chronic illnesses [6, 8] and are recommended by regulatory agencies such as the Food and Drug Administration and the European Medicine Agency, to support the approval of new therapies and medical labelling claims [9, 10].

Congenital disorders of glycosylation (CDG) are a growing family of rare diseases that affect the synthesis and attachment of sugar ‘trees’ (glycans) of proteins and lipids. These defects often have severe, multi-organ implications for the patients, since about 50% of human proteins are glycosylated and glycans play essential roles in all biological processes [11]. PMM2-CDG is the most common CDG, and it is due to autosomal recessive variants in the PMM2 gene, which encodes the enzyme phosphomannomutase 2, essential for N-glycosylation. This enzyme is responsible for the synthesis of N-linked oligosaccharides by converting mannose 6-phosphate to mannose 1-phosphate [12]. PMM2-CDG clinical presentation is dominated by neurologic abnormalities such as psychomotor disability, seizures, hypotonia and ataxia, besides multiple organ involvement resulting in chronic disability, poor QoL and premature death [13]. Some potential treatments, such as liposome-encapsulated mannose 1-phosphate administration, are undergoing clinical studies [14]. More recently, a trial with acetazolamide showed improvement of the ataxia [15]. Moreover, in a single-patient paediatric trial with epalrestat, improvements in ataxia and also in growth were observed [16]. However, specific tools are needed to measure QoL in PMM2-CDG to understand if a treatment has a significant impact.

Currently, there are no disease-specific QoL PROMs/ObsROMs for PMM2-CDG. Here, we used a community-centric approach, involving CDG medical professionals and families in the design and conduction of the study. We aimed to gather PROMs and ObsROMs that are specific for the most impactful PMM2-CDG clinical signs and symptoms. For that purpose, we surveyed PMM2-CDG families and clinicians following PMM2-CDG patients to understand which are the most onerous signs and symptoms, and interviewed families to understand the real burden of those clinical manifestations in everyday life. Considering the input of these stakeholders, we reviewed the literature about the PROMs and ObsROMs used in other rare and common diseases with similar signs and symptoms to PMM2-CDG. Those tools could potentially be validated and applied directly to the PMM2-CDG population or adapted to create the first PMM2-CDG-specific QoL questionnaire.

## Methods

1. Set up of the patient and medical advisory committees

Two advisory committees were established to provide expert insights regarding the understanding and particularities of the disease and to guide decision making throughout this project. Patient experts, specifically 11 family caregivers, and 9 medical experts were invited to participate in the committees. A summary of the project and an explanation of their roles were provided if they agreed to participate. Communications were mainly done by email or by video calls when necessary.

2. Quantitative analysis of PMM2-CDG symptoms’ impact (PMM2-CDG Symptoms’ Impact Survey)

A survey was constructed to assess the impact of the signs and symptoms from infancy to adulthood. Two versions were used, one targeting PMM2-CDG families and the other targeting medical experts. Electronic samples of the survey are available at https://www.surveymonkey.com/r/HCPCOM (medical experts’ version) and https://www.surveymonkey.com/r/PATCOMM (version adapted to families). The survey included an exhaustive list of signs and symptoms reported in the OMIM database (MIM: 212065) but also reported by CDG families. Family experiences included both personal communications and social media reports in the CDG Global Alliance Facebook Group, a social media platform uniting worldwide CDG patients and professionals perceived as a safe environment where the community openly shares questions, concerns, and experiences. The information derived from this group complies with the terms and conditions of the platform and with the privacy settings of the participants. It was shared in a voluntary manner with all participants of the group and fell under the objectives of the group (i.e., promoting shared knowledge between families, doctors, and researchers). This was a complementary step to validate and complete the information collected through other sources, therefore, the information was not transcribed and thus is not traceable ans constitute no risk of harm to the participants. Anonymity was maintained in all instances. Printed surveys were distributed at the beginning of the 4th World Conference on CDG for Families and Professionals, held in Lisbon on the 26th and 27th July 2019. Given that most PMM2-CDG patients are unable to provide self-reports due to the fact that (1) most are of paediatric age and (2) have considerable cognitive impairment, patients' views were evaluated and conveyed by patients' families. Observer and proxy reports have been commonly used in studies where self-reports cannot be obtained [17–19]. Therefore, patients’ caregivers answered the survey voluntarily following written and verbal information about the study. Respondents were asked to classify the daily life impact of each of the symptoms/clinical manifestations on a scale of 1—“No impact” to 5—“Extremely negative impact” considering each phase of the patient’s life (infancy: 0–3 years; childhood: 4–10 years; adolescence: 11–17 years; and adulthood: 18 years and older). To increase data collection, respondents could answer to more than one age range as long as they felt comfortable and confident in doing so (e.g., the caregiver of an adolescent patient could answer both the infancy, childhood and adolescent sections). An “I don't know/cannot answer” option was available to improve data collection and quality. Additionally, respondents were given the chance to share relevant information that they felt was missing in the survey by including an optional text field: “If there are other symptoms you find impactful, please list them here and rate the magnitude of their impact (using the same scale)”. The surveys were collected by the end of the conference. The final impact level of each clinical manifestation was calculated using the mean value of all respondents for each given age range. The 7 symptoms with higher impact level for each age range for both families and professionals were summed up, yielding a final list of 16 unique impactful symptoms (7 symptoms × 4 age ranges × 2 target groups = 56–40 duplicates = 16 unique symptoms). To analyse the differences between the families’ and clinicians’ perspectives, for each sign/symptom, a two-way ANOVA test with multiple comparisons and Sidak’s correction was performed yielding an adjusted p-value. Statistical significance was considered if adjusted p-value < 0.05.

3. Qualitative insights of PMM2-CDG symptoms’ impact

Interviews were designed and led to gather insights about the real-word impact of the signs and symptoms identified in the survey as being “the most impactful” from families’ perspectives (Additional file 1: Table 1). All medical and difficult terms were referred to in lay-language and further explained when required by the participant to ensure their understanding. Deidentified transcripts were obtained from seven interviews of mothers of PMM2-CDG patients which were part of our patient committee. Demographics of the patients included in the interviews are available in Additional file 1: Table 2. The interviewees were prompted to share patient experiences in greater depth. Hence, questions were open-ended to avoid bias and were not read verbatim to permit free-flowing discussion. The collected insights were used to guide our article selection to make it more specific and targeted to the patient’s needs.

**Fig. 1:**
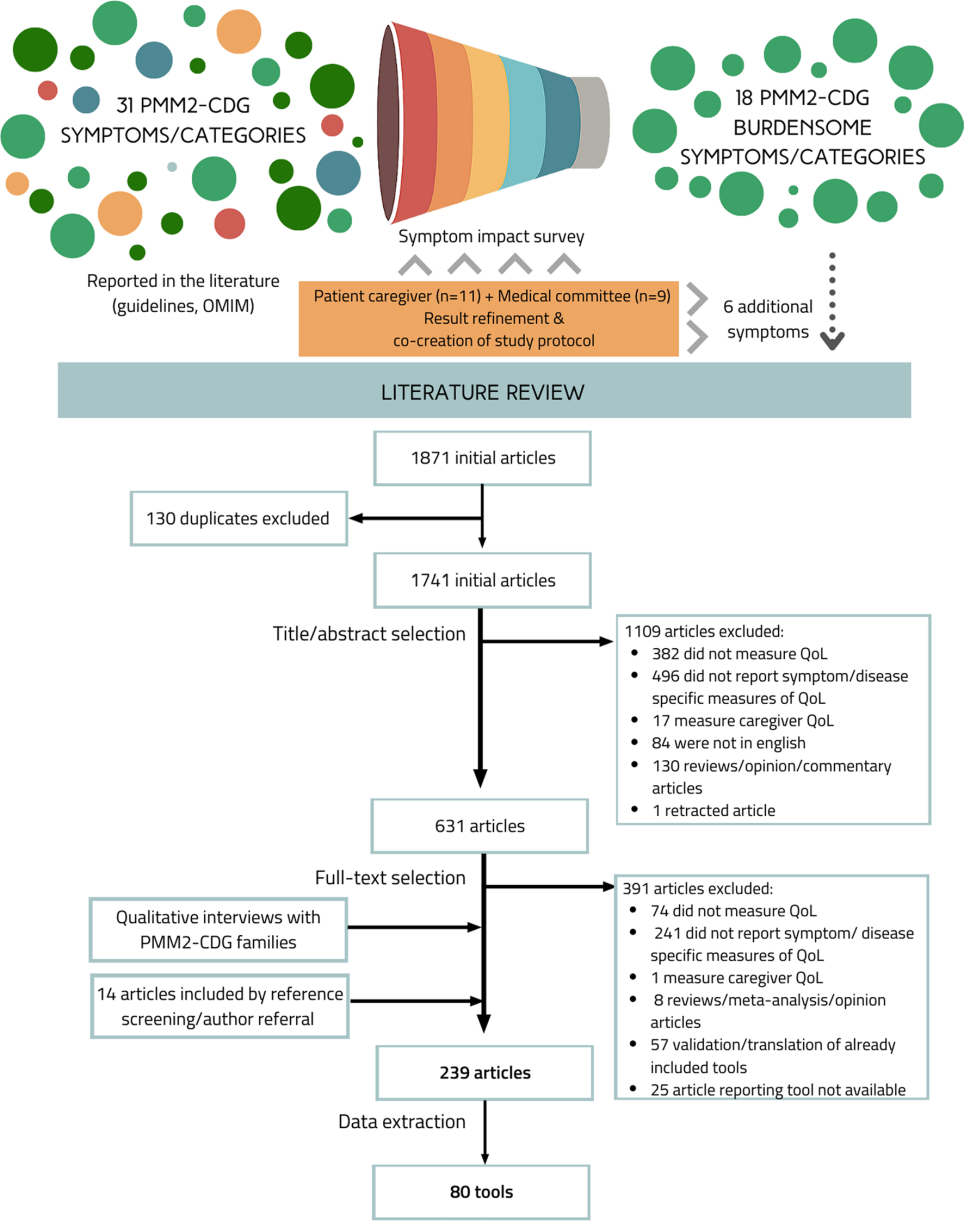
Overview of the study workflow

4. Review of the literature
  - Search strategy
    The community-identified burdensome signs ans symptoms guided a literature review strategy to identify and gather specific PROMs and ObsROMs. The PubMed database was queried with pre-defined search terms on September 11th, 2020. The search query was based on three groups of search terms: (1) QoL related, (2) PROMs/ObsROMs related terms, and (3) impactful signs and symptoms previously identified—connected by the Boolean operator “AND” (Additional file 1: Table 3). Keywords within the same group were connected using the operator “OR”. For some signs and symptoms and given the fact that PMM2-CDG is a rare metabolic disorder, the keywords “metabolic” or “rare disease” were added to the combination to provide more specific results. Resulting articles from the search were exported and duplicated articles were eliminated. References of relevant articles were screened, and additional articles were included by author referral (Fig. 1).
  - Study selection and data extraction
    Resulting articles were screened based on predefined inclusion and exclusion criteria. Studies had to be written in English and measure HrQoL for one or more of the previously identified impactful signs/symptoms, by means of a PROM or ObsROM. Articles using clinician-reported outcomes, performance outcomes, interviews and reviews were excluded. Furthermore, studies reporting caregiver QoL and that explicitly affirmed the use of non-English translations of the PROMs/ObsROMs were excluded. Nevertheless, articles describing the use of foreign (non-English) questionnaires for which an English translation is available were included (e.g., Deglutition Handicap Index, Izumo scale). Article titles and abstracts were screened and, subsequently, the full-text versions of the remaining articles were evaluated according to the inclusion/exclusion criteria (Fig. 1). Article content analysis and data extraction was performed by a group of 4 researchers, specifically regarding the PROMs employed to measure the QoL, the participants' cohort and disease(s)/sign(s)/symptom(s) assessed. For some sign(s)/symptom(s), no specific tool was found. In these cases, some adequate items or subscales were secondarily captured by the inclusion of other tools.
  - Quality analysis

**Table 1:**
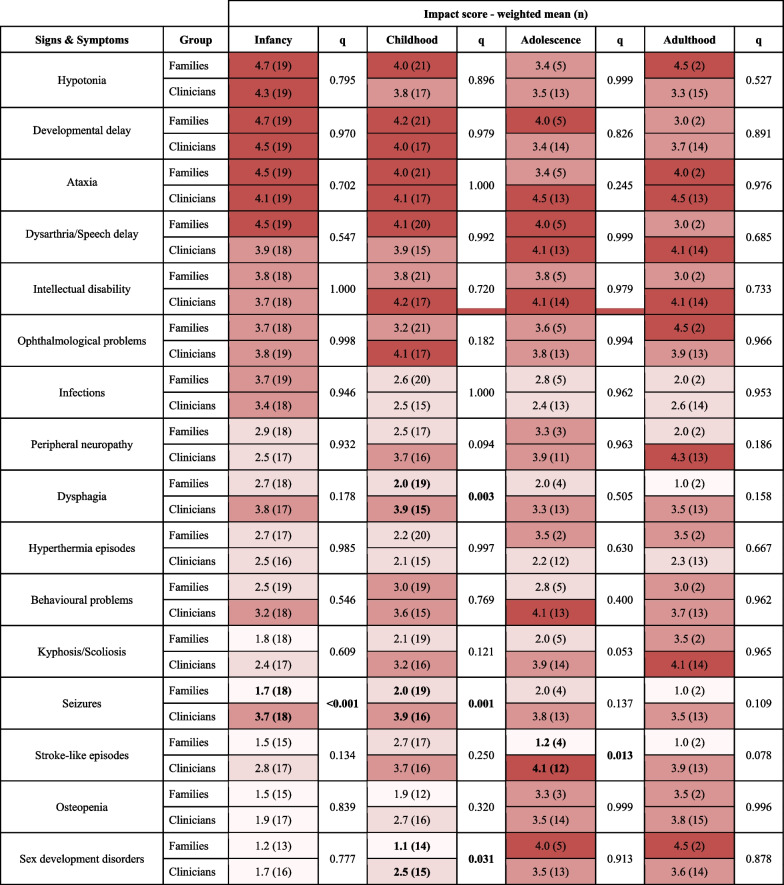
Impact scores (n) for selected signs and symptoms by age range and according to families and clinicians’ perspectives

1– < 2—No or slight negative impact; 2– < 3—Moderate negative impact; 3– < 4—Negative impact; 4– < 5—Extremely negative impact. q—adjusted p-value

**Table 2:** Summary of the characteristics of included articles

Article summary	N	%
*Number of patients*		
≤ 100	136	56.9
101 to 500	73	30.5
≥ 501	30	12.6
*Age Range*		
Pediatric (< 18)	23	9.6
Adult (≥ 18)	187	78.2
Both	29	12.1
*Type of QoL report*		
Self-reported	226	94.6
Proxy-reported	4	1.7
Both	9	3.8
*Disease classification (ICD-11)*		
Neoplasms	7	2.9
Diseases of the blood or blood-forming organs	4	1.7
Diseases of the circulatory system	60	25.1
Diseases of the immune system	2	0.8
Endocrine, nutritional or metabolic diseases	5	2.1
Sleep–wake disorders	1	0.4
Diseases of the nervous system	18	7.4
Diseases of the visual system	58	24.3
Diseases of the respiratory system	3	1.3
Diseases of the digestive system	58	24.3
Diseases of the musculoskeletal system or connective tissue	15	6.3
Diseases of the urinary system	1	0.4
Developmental anomalies	1	0.4
Symptoms, signs, or clinical findings, not elsewhere classified	6	2.5
Total number of included articles	239	

The sum of the percentages might not yield 100% due to numerical rounding

**Table 3:** Symptom/disease-specific quality of life tools per impactful symptom/category

Category	Instrument name	Subscale/domains	No. items	Target Group (age, years)	*Diseases assessed*	*Ref*
*Ataxia*	Quality of Life in Essential Tremor Questionnaire (QUEST)	Communication, work and finances, hobbies and leisure, physical, psychosocial	30	≥ 18	Essential tremor	[37]
	Multiple System Atrophy Quality of Life questionnaire (MSA-QoL)	Motor, nonmotor, emotional/social functioning	40	≥ 18	Multiple system atrophy	[38]
*Cardiomyopathy*	Kansas City Cardiomyopathy Questionnaire (KCCQ-12)	Physical limitation, symptom frequency, quality of life, and social limitation	12	No reference	Cardiomyopathy	[39]
	Minnesota Living with Heart Failure Questionnaire (MLHFQ)	Physical impairment, emotional impairment	21	No specification ("elderly")	Heart failure	[40, 41]
*Coagulopathy*	Haemophilia-specific health-related quality of life questionnaire for adults (HAEMO-QoL-A)	Physical functioning, role functioning, worry, consequences of bleeding, emotional impact, treatment concerns	41	≥ 18	Haemophilia	[42]
	Haemophilia-specific health-related quality of life questionnaire—short form (HAEMO-QoL-SF)	Physical health, feelings, view of yourself, family, friends, other people, sports & school, dealing with haemophilia and treatment	35	4–17 (4–7; 8–17)	Haemophilia	[43]
	Canadian Haemophilia Outcomes-Kids’ Life Assessment Tool (CHO-KLAT)	Treatment, physical health, family, future, feelings, understanding of haemophilia, other people and friends, and control over your life	35	4–18	Haemophilia	[44]
*Dysarthria*	Quality of Life in the Dysarthric Speaker (QOL-Dys)	Speech characteristics of the word, situational difficulty, compensatory strategies, perceived reactions of others	40	≥ 18	Dysarthria	[45, 46]
	Living with Neurologically Based Speech Difficulties (LwD)	Comunication problems related primarily to speech, comunication problems related primarily to language/cognition, comunication problems related primarily to fatigue, effects of emotions, effects of different persons, effects of different situations, consequences of my difficulties in communicating, what contributes to the changes in the ways I communicate, communicating like I would want to, how do I perceive changes and the possibility to alter my ways of speaking	50	≥ 18	Parkinson disease	[47]
	Dysarthria impact profile (DIP)	The effects of dysarthria on me as a person; accepting my dysarthria; how i feel others react to my speech; how my dysarthria affects my communication with others	52	No reference	Dysarthria acquired through different diseases (e.g. multiple sclerosis, motor neuron discase, Parkinson disease, stroke, Friedreich ataxia)	[48]
	Speech Handicap Index (SHI)	Psychosocial function; speech function	30	No reference	Oral and pharyngeal cancer	[49]
*Dysphagia*	Swallowing Quality Of Life (SWAL-QOL)	Burden, eating duration, eating desire, food selection, communication, fear, mental health, social, fatigue, and sleep	44	≥ 18	Friedreich ataxia; laryngotracheal disease; dysphagia	[50–52]
	Deglutition Handicap Index (DHI)	Physical, functional and emotional	30	≥ 18	Dysphagia	[53, 54]
	Eating Assessment Tool (EAT-10)	Unidimensional	10	≥ 18	Dysphagia	[55, 56]
	Dysphagia Handicap Index (DHI)	Physical, emotional, and functional problems	25	≥ 18	Dysphagia	[57]
*Food Allergy*	Food Allergy Quality of Life Questionnaire—Parent Form (FAQLQ-PF)	Emotional impact, food anxiety, social/dietary limitations	30	0–12 (0–3; 4–6; 7–12)	Food allergy	[58]
	Celiac Disease-specific DUX questionnaire (CDDUX)	Communication, having celiac disease, scale diet	12	8–18 (or their caregivers)	Celiac disease	[59]
*Gastrointestinal problems*	Short Inflammatory Bowel Disease Questionnaire (SIBDQ)	Bowel symptoms, systemic symptoms, emotional functioning, social functioning	10	≥ 18	Inflammatory bowel disease	[60]
	Inflammatory Bowel Disease Questionnaire (IBDQ)-9	Total score	9	≥ 18	Inflammatory bowel disease	[61]
	Irritable Bowel Syndrome Quality of Life Questionnaire (IBSQoL)	Emotional health, mental health, health belief, sleep, energy, physical functioning, diet, social role, physical role, and sexual relations	30	≥ 18	Irritable bowel syndrome	[62]
	Irritable Bowel Syndrome Quality of Life (IBS-QOL)	Dysphoria, interference with activity, body image, health worry, food avoidance, social reaction, sexual, relationships	34	≥ 18	Irritable bowel syndrome, chronic functional constipation, inflammatory bowel disease	[63, 64]
	Gastroesophageal Reflux Disease-Health Related Quality of Life (GERD-HRQL)	Total score	10 + 1 not scored	No reference	Gastroesophageal reflux disease	[65]
	GastroIntestinal Quality of Life Index (GIQLI)	Core symptoms, physical items, psychological items, social items, disease-specific items	36	≥ 18	Gastrointestinal problems; type 2 diabetes mellitus; seveve obesity; post-Whipple surgery and postcholecystectomy	[66]
	Scleroderma gastrointestinal tract 1.0 questionnaire (SSC-GIT 1.0)	Reflux/indigestion, diarrhea, constipation, pain, emotional well-being, social functioning	52	≥ 18	Systemic sclerosis	[67]
	EORTC QLQ—Oesophageal Module 18 (OES18)	Dysphagia, eating, reflux, and pain plus 6 single items (swallowing saliva, choking, dry mouth, taste, cough, speech)	18	≥ 18	Dysphagia, esophageal eosinophilia, esophageal cancer	[68]
	EORTC QLQ- Gastrointestinal Neuroendocrine Carninoid Module (GINET21)	Endocrine symptoms, GI symptoms, treatment-related symptoms, social functioning and disease-related worries plus 4 single-items (muscle and/or bone pain, body image, information and sexual functioning)	21	≥ 18	Gastroenteropancreatic neuroendocrine tumours	[69, 70]
	Home parenteral nutrition-quality of life (HPN-QOL)	General health, ability to holiday or travel, coping, physical function, ability to eat and drink, employment, sexual function, and emotional function, body image, immobility, fatigue, sleep pattern, gastrointestinal symptoms, other pain, presence or absence of a stoma, financial issues, and weight	47	≥ 17	Chronic intestinal failure patients treated with home parenteral nutrition	[71, 72]
	Izumo scale for abdominal symptom-related QOL	Heartburn, gastralgia, postprandial fullness, constipation, diarrhea	15	No reference	Type 2 diabetes mellitus; upper gastrointestinal symptoms, bone resorption and back pain in postmenopausal osteoporosis patients	[73]
	Short Form-Nepean Dyspepsia Index (SF-NDI)	Interference with daily activities, knowledge/control, tension, work/study and eating/drinking	10	≥ 18	Irritable bowel syndrome	[74]
	IBDQ-32	Bowel symptoms, systemic symptoms, emotional function and social function	32	≥ 18	Irritable bowel syndrome, irritable pouch syndrome and inflammatory bowel disease (Crohn disease and ulcerative colitis)	[75]
	IBDQ-36	Bowel symptoms, systemic symptoms, functional impairment, emotional functioning, social impairment	36	≥ 18	Inflammatory bowel disease	[76]
*Infections*	Sino-Nasal Outcome Test (SNOT-22)	Rhinologic, extra-nasal rhinologic, and ear/facial symptoms, psychological and sleep dysfunction	22	≥ 16	Chronic rhinosinusitis	[77]
	Sino-Nasal Outcome Test (SNOT-20)	Rhinologic, extra-nasal rhinologic, and ear/facial symptoms, psychological and sleep dysfunction	20	≥ 18	Bronchiectasis, allergic fungal rhinosinusitis	[78]
	Leicester Cough Questionnaire (LCQ)	Physical, psychological and social	19	≥ 18	Bronchiectasis	[79]
*Kyphosis/Scoliosis*	Scoliosis Research Society (SRS-30)	Function/activity, pain, self-image/appearance, mental health, satisfaction with management	31	13–17, 18–64	Scoliosis	[80]
	SRS-22; SRS-22r	Function/activity, pain, self-image/appearance, mental health, satisfaction with management	22	13–17; 18–64	Adolescent idiopathic scoliosis	[81, 82]
	SRS-7	Unidimensional	7	13–18	Adolescent idiopathic scoliosis	[83]
	Early-Onset Scoliosis Questionnaire (EOSQ-24)	General health, pain/discomfort, pulmonary function, transfer, physical function, daily living, fatigue/energy level, emotion, parental impact, financial impact, satisfaction	24	0–18	Early-onset scoliosis	[84]
	Scoliosis Quality of Life Index (SQLI)	Physical activity performance, back pain, self-esteem, moods & feelings, and satisfaction with management	22	10–18	Adolescent idiopathic scoliosis	[85]
	The Italian Spine Youth Quality of Life questionnaire (ISYQOL)	Unidimensional	20	10–18	Adolescent idiopathic scoliosis	[86, 87]
*Liver problems*	Chronic Liver Disease Questionnaire for NAFLD and NASH (CLDQ NAFLD/NASH)	Abdominal symptoms, activity/energy, emotional health, fatigue, systemic symptoms, and worry	36	≥ 18	Non-alcoholic steatohepatitis	[88, 89]
	Chronic Liver Disease Questionnaire (CLDQ)	Fatigue, activity, emotional function, abdominal symptoms, systemic symptoms, and worry	29	≥ 18	Cirrhosis, hepatic encephalopathy, cholestatic liver diseases	[90]
*Ophthalmological problems*	National Eye Institute Visual Functioning Questionnaire—25 (NEI VFQ-25)	General vision, near vision, distance vision, driving, peripheral vision, color vision, ocular pain, general health, and vision-specific role difficulties, dependency, social function, and mental health	25	≥ 18	RLBP1 retinitis pigmentosa, Friedreich’s ataxia, myopia, hyperopia, astigmatism, nystagmus, strabismus, spinocerebellar ataxia, glaucoma	[91]
	National Eye Institute Refractive Error Quality of Life Instrument—42 (NEI RQL-42)	Clarity of vision, expectations, near vision, far vision, diurnal fluctuations, activity limitations, glare, symptoms, dependence on correction, worry, suboptimal correction, appearance, and satisfaction with correction	42	≥ 18	Myopia, astigmatism, hyperopia	[92]
	VFS-plus	Unidimensional	19	≥ 18	Retinitis pigmentosa	[93]
	Pediatric Eye Questionnaire (PedEyeQ)	Functional vision, bothered by eyes/vision, social, frustration/worry, eye care	39–42	< 18 (0–4; 5–11; 12–17)	Strabismus and anisometropia	[94]
	Intermittent Exotropia Questionnaire (IXTQ)	Unidimensional	12	5–17 (5–7; 8–17)	Intermittent exotropia, strabismus, children wearing spectacles	[95]
	Adult Strabismus Quality of Life Questionnaire (AS-20)	Psychosocial and function	20	≥ 18	Epiretinal membrane, nondiplopic childhood-onset strabismus, strabismus, glaucoma, diplopia	[96]
	Adult Strabismus Quality of Life Questionnaire—11 item (AS-11)	Unidimensional	11	≥ 18	Strabismus	[97]
	Low Luminance Questionnaire (LLQ)	Driving, extreme lighting, mobility, emotional distress, general dim lighting, peripheral vision	32	≥ 18	RLBP1 Retinitis Pigmentosa	[98]
	Questionnaire on the impact of strabismus on patient quality of life based on AS-20 from Ribeiro Gde B et al., 2014	Psychosocial aspects and functional aspects	20	> 7	Strabismus	[99]
	College of Optometrists in Vision Development Quality of Life questionnaire (COVD QOL)	Somatic, physical/occupational, social, and psychological	30	> 7	Intermittent central suppression of vision	[100]
	8-question QoL live interview developed by Kothari M et al., 2009	No reference	8	4–16	Strabismus	[101]
	Vision Quality of Life Questionnaire by McKeon C et al., 1997	Psychological well-being, perception of health, role functioning, physical health, visual function	32	8–46	Intermittent exotropia	[102]
	Questionnaire for Evaluating Quality of Life of Pathologic Myopia Patients by Takashima T et al., 2001	Vision-related daily tasks, social handicaps, emotional handicaps, leisure and support, cognition about disease, general well-being schedule, eye satisfaction, life satisfaction	52	≥ 18	Myopia	[103]
	Amblyopia and Strabismus Questionnaire (A&SQ)	Distance estimation, visual disorientation, fear of losing the better eye, diplopia, and social contact and cosmetic problems	26	≥ 18	Amblyopia, small-angle diplopia, strabismus	[104]
	Quality of Life Impact of Refractive Correction (QIRC)	Visual function, health concerns, well-being, convenience issues, symptoms, economic issues and cognitive issues	20	≥ 16	Myopia	[105]
	10-item Neuro-Ophthalmic Supplement (NOS) to the NEI-VFQ-25	Visual function and eye/lid appearance	10	≥ 18	Spinocerebellar ataxia	[106]
	HRQOL questionnaire by Chen et al., 2015	Visual function, psychosocial impact, social interaction and worries about vision	16	7–12	Amblyopia	[107]
	Disease specific questionnaire based on the study of the Joint LASIK Study Task Force (Schallhorn et al., 2016)	Patient satisfaction, effect of vision on various activities, ocular discomfort and visual phenomena	23	≥ 18	Post laser in situ keratomileusis intervention	[108–110]
	Nystagmus-specific QOL questionnaire (NYS-29)	Personal and social functional and physical and environmental functional	29	≥ 18	Nystagmus	[111]
	Quality-of-Life Scale for Myopia by Erickson et al., 2004	Tolerance of compromise/symptom, psychological states, frequency of compromise/symptom, extraversion/introversion, cosmesis	52	≥ 18	Myopia	[112]
*Osteopenia*	Short Osteoporosis Quality of Life Questionnaire (ECOS-16)	Physical functioning, illness-related fears, psychosocial functioning, and pain	16	≥ 18	Postmenopausal osteoporosis	[113, 114]
	Osteoporosis Assessment Questionnaire—Short Version (OPAQ)	Physical function, emotional status, symptoms and social interaction	34	≥ 18	Postmenopausal osteoporosis	[115]
	Osteoporosis-Targeted Quality of Life (OPTQoL)	Physical difficulty, adaptations and fears	32	≥ 18	Female osteoporosis	[116]
	Osteoporosis Quality of Life Questionnaire (OQLQ)	Symptoms, physical function, activities of daily living, emotional function and leisure	30	≥ 18	Female osteoporosis	[117]
	Mini-Osteoporosis Quality of Life Questionnaire (Mini-OQLQ)	Symptoms, physical function, activities of daily living, emotional function and leisure	10	≥ 18	Postmenopausal osteoporosis	[118]
	Quality of Life in Osteoporosis (Qualiost)	Physical and emotional	23	≥ 18	Osteoporosis	[119]
*Seizures*	Epilepsy & Learning Disabilities Quality of Life Questionnaire (ELDQOL)	Behavior, seizure activity, mood and side effects	70	0–18	Epilepsy and learning disabilities, Dravet syndrome	[120, 121]
	Quality Of Life In Epilepsy (QOLIE-10/QOLIE-10-P)	Epilepsy effects, mental health, role function	10/11	≥ 18	Epilepsy	[122]
	Quality Of Life In Epilepsy (QOLIE-31)	Seizure worry, overall QOL, emotional wellbeing, energy/fatigue, cognitive functioning, medication effects, social functioning	31	≥ 18	Epilepsy	[123]
	Quality of Life in Epilepsy Inventory for Adolescents (QOLIE-AD-48)	Epilepsy impact, memory-concentration, attitudes towards epilepsy, physical function, stigma, social support, school behavior, health perception	48	11–17	Epilepsy	[124]
	Quality of Life in Childhood Epilepsy Questionnaire (QOLCE)	Physical restrictions, energy/fatigue, attention/concentration, memory, language, other cognitive processes, depression, anxiety, control/helplessness, self-esteem, social interactions, social activities, stigma, behaviour, general health, and quality of life	91	4–18	Epilepsy	[125]
	Quality of Life in Childhood Epilepsy Questionnaire (QOLCE-55)	Cognitive, emotional, physical and social functioning	55	4–12	Epilepsy	[126]
*Sleep disturbances*	Insomnia Severity Index (ISI)	Initial (sleep-onset), middle (sleep maintenance), terminal (awakening), satisfaction, interference (with daily functioning), noticeability and distress	7	≥ 18	Insomnia	[127]
	Pittsburgh Insomnia Rating Scale (PIRS)-20	Nighttime and daytime distress symptoms, sleep parameters, quality, regularity, and depth of sleep	20	≥ 18	Diabetes mellitus type II and insomnia	[128]
*Several signs/symptoms*	Cushing QoL Questionnaire (CushQoL)	Daily life, emotional, and physical aspects	12	≥ 18	Cushing syndrome	[129]
	University of Washington Quality of Life Questionnaire (UW-QOL)	Pain, appearance, activity, recreation, swallowing, chewing, speech, shoulder, taste, saliva, mood and anxiety	12	≥ 18	Head and neck cancer	[130]
	University of California Los Angeles Prostate Cancer Index (UCLA-PCI)	Urinary, sexual and bowel function, urinary, sexual and bowel bother	20	≥ 18	Early state of prostate cancer	[131]

The purpose of the quality analysis was not to perform a systematic review of the psychometric properties of the included instruments, but rather to identify and compare them in terms of their psychometric properties, namely Content, Criterion and Construct Validity, Internal Consistency, Agreement, Reliability, Responsiveness, Floor and Ceiling effect and Interpretability (Additional file 3). To do so, this analysis was based on the original development and/or validation articles of the instruments. Thus, translations or validations to other languages besides English were not considered. One instrument could not be evaluated (Scoliosis Research Society-30) as its development and validation articles were not available. Also, three instruments (Short Inflammatory Bowel Disease Questionnaire, Pittsburgh Insomnia Rating Scale, and College of Optometrists in Vision Development Quality of Life Questionnaire) were evaluated exclusively based on the available abstracts.

The analysis was made using the Quality Criteria for Measurement Properties of Health Status Questionnaire developed by Terwee et al., (2007) for the design, methods, and outcomes of the development and validation studies [20]. Based on these criteria, each psychometric property was evaluated with (+)—positive rate; (?)—indeterminate or doubtful rate; (–)—negative rate; or (0)—no information available. Some adaptations of the criteria were needed:

1. for Construct Validity evaluation, the criteria for a positive rating requires that specific hypotheses have been formulated and at least 75% of the results are in accordance with them. However, given that for the majority of the articles, hypotheses were not explicitly presented by the authors, we had to analise if the goal of development and/or validation of the instrument was met;
2. for Internal Consistency, the criteria of the sample size being N = 7 $\times$ the number of items and N > 100 was not considered for two reasons. First, because of the great variability in the number of items between questionnaires, and second, because we are dealing with symptom/condition-specific questionnaires, and therefore, the clinical samples of validation articles are usually smaller than if we were dealing with an healthy population.;
3. when in doubt about meeting less objective criteria (e.g., *convincing arguments that gold standard is ‘‘gold’’*, for Criterion Validity), the properties were classified with a positive rating if the validation methodology was clearly described and the authors clearly justified their conclusions well.

## Results

1. Selection of the most impactful symptoms by the community and the expert committees
  The PMM2-CDG symptoms’ impact survey had 42 respondents: 23 family representatives and 19 PMM2-CDG medical experts. A list of the topmost impactful symptoms was then obtained considering the sum of the 7 most impactful manifestations across the 4 age ranges considered according to both families and clinicians and excluding duplicates. This resulted in a list of 16 signs and symptoms (Table 1).
  There was a good level of agreement between the perspectives of families and clinicians, particularly for the infancy period. For this age group, only seizures were rated with a statistically significant difference (q < 0.001) between families (IS = 1.78, n = 18) and clinicians (IS = 3.67, n = 18). Significant differences between the views of both stakeholders were predominant for the childhood group. During this timeframe, dysphagia (IS = 2.00, n = 19 for families and IS = 3.93, n = 15 for clinicians, q = 0.003) and seizures (IS = 2.05, n = 19 for families and IS = 3.87, n = 16, q = 0.001) were perceived to have a much higher negative impact by clinicians while for families a moderate negative impact was reported. The presence of sex development issues was also associated with a bigger impact by the medical doctors (IS = 2.53, n = 15) in comparison to PMM2-CDG families (IS = 1.14, n = 14; q = 0.031). Concerning stroke-like episodes, an extremely negative impact on adolescent PMM2-CDG patients was perceived by clinicians (IS = 4.08, n = 12) while none to slight negative impact (IS = 1.25, n = 4) were alleged by family members (q = 0.013). The same tendency was seen for the adult group. Lastly, although not statistically significant, clinicians tended to rate kyphosis/scoliosis with a more pronounced negative impact than families during adolescence and childhood. The same happened regarding peripheral neuropathy, particularly in the childhood and adulthood group (Table 1).
  The analysis of the qualitative data shared on the survey as well as the revision of the most impactful signs and symptoms by the family and medical committees resulted in the inclusion of 6 additional clinical manifestations, namely sleep disturbances, liver problems, coagulopathy, food allergies, cardiomyopathy, and pericardial effusion.
2. Families’ perspectives about the real-world impact of the most impactful symptoms
  Semi-structured interviews with open-ended questions were led with family members of PMM2-CDG patients which allowed them to express the burden of living with the disease and the consequences of specific clinical manifestations in family life. The summary results of the interviews encompassing the experiences with the complete list of clinical manifestations are described in Additional file 2. This information allowed us to refine and further tailor our article and QoL assessment tools selection to the experiences of PMM2-CDG families. As an example, osteopenia/osteoporosis, clinically characterized by low bone density, occurs in PMM2-CDG patients at a later stage in life, but with significant consequences for the patient's daily life. One family member stated that “[osteoporosis] *causes pain when she is sitting in the wheelchair as well as getting up and sitting down. We are afraid of bone fractures so we avoid physical activities and falls as they are frightening. (…) She is being treated every 6 months at the hospital with a bone cancer treatment which has a lot of side effects during 5 days. She is in a frustrated state, with fever, pain to touch, she can’t move and is incontinent*” (mother of a 40 years-old PMM2-CDG patient). In another experience, having osteopenia/osteoporosis limits the management of other clinical manifestations: “*due to osteopenia, he can’t have surgery of the scoliosis because of the bone fragility. He cares about it* [scoliosis] *when he is in the wheelchair because it is very noticeable. There is not enough space on his body for the intestines and his lungs and sometimes he has very fast and short breathing*” (mother of a 25 years-old PMM2-CDG patient). This guided the QoL tools selection by making sure osteopenia/osteoporosis specific tools included items referring to pain, fear of fractures/falls, self-image, impact in care or treatment impact.
3. Review of the literature results according to the community-selected symptoms real-world qualitative information
  The review of the literature concerning the application of PROMs specific for the community-selected impactful PMM2-CDG symptoms/manifestations resulted in the inclusion of 239 articles (Fig. 1). The characteristics of the included articles are summarized in Table 2. Most articles (58.1%) included small participant cohorts of ≤ 100 participants. While 29.9% reported cohorts of > 100 to < 500 participants, only 12% of the studies reported more than > 500 participants. Studies of adult populations represent most of the included studies (78%). Only 10% of the included studies focused on pediatric populations and 12% included both adult and pediatric populations. QoL self-reports were described by most studies (94.2%) whilst proxy-reports or a combination of both accounted for 2.1% and 3.7% of the studies, respectively. Among the included articles, 14 disease groups were represented. Particularly prevalent in our study sample were diseases of the digestive, visual, and circulatory system followed by diseases of the musculoskeletal system, the connective tissue, and the nervous system.
  The review of the included articles allowed the identification of 80 tools. These tools were grouped by signs/symptoms in Table 3. The list of references supporting the inclusion of such tools can be found in Additional file 1: Table 4. From the 22 groups of signs and symptoms, specific QoL tools were found for 15 of them (Table 3). No specific tools were found for 7 of the most impactful clinical manifestations, particularly for developmental delay, intellectual disability, hypotonia, pericardial effusion, peripheral neuropathy, stroke-like episodes, or symptoms related to deficient sexual development. However, even though no specific instruments were found for behavior, developmental or intellectual problems, other included tools specific for other symptoms/diseases include subscales or items specific for those areas (e.g., mood swings, depression, physical, mental, and social functioning, etc.).
4. Quality assessment of included questionnaires

The quality of the 80 instruments was analyzed using specific criteria from Terwee et al. (2007) (Additional File 3) [20]. Most instruments were evaluated with positive rating ( +) for Content Validity (93.7%), Construct Validity (77.5%), Internal Consistency (71%) and Reliability (60.8%). For Agreement (73.4%), Floor and Ceiling Effect (67.1%), and Responsiveness (55.7%), no sufficient information was found for most of the instruments. Lastly, regarding Criterion Validity and Interpretability analysis, the greater part of the information was unavailable (35.4% and 24%, respectively) or indeterminate (22.5% and 59.4%, respectively).

## Discussion and future perspectives

Patient-centered outcomes have gained recognition in health technology assessment and clinical trial settings. Besides, they provide unique insights into the disease’s natural history in terms of QoL and its fluctuations over time. Rather than just measuring clinically important outcomes, they offer the opportunity to access “patient-important” outcomes, meaningful to them when evaluating treatments or care [21]. For complex, chronic and/or rare diseases—with holistic challenges and for which the definition of disease biomarkers or clinical endpoints is puzzling—patient-reported QoL is of major importance providing a direct interpretation of the patient’s response to treatment or care [22]. However, the scarcity of valid QoL PROMs and ObsROMs for most rare diseases and the challenges of validating the current available tools, pose a problem to adequately appraise potential treatments. Creative and pragmatic solutions are warranted to overcome difficulties related to small patient cohorts, the cost of tools’ development, and the urgency for making new therapies available [4, 5].

In this study, we applied an innovative methodology to accelerate the development of a PMM2-CDG-specific QoL questionnaire, while assuring its adequacy and meaningfulness by including the views and experiences of families and medical experts. By including both stakeholders’ quantitative and qualitative input in the design of our literature search, we identified QoL instruments that matter the most. The differences in the perception of the most burdensome signs and symptoms between patients/caregivers and clinicians underscore patient/caregiver engagement and participation in all stages of the development of PROMs or ObsROMs as the only way to safeguard the relevance, adequacy, and comprehensibility of these tools [23]. However, particularly in rare, heterogeneous diseases, complementing the individual experience with the knowledge of medical experts is critical. While patients and family caregivers can highlight “hidden” aspects of the disease that are not clear or do not seem important to doctors, the latter can provide a wider perspective on the frequency, severity, and impact of clinical manifestations by studying patient cohorts. Furthermore, clinicians will consider potential disease complications that patients may have not yet experienced. Importantly, conducting qualitative interviews complemented the quantitative results on “What is more important?” with “How and why is it more important?”. In other words, listening to the description of the patient/family experience with illness (i.e., narrative medicine) provides meaning and understanding but also identifies the real impact of the disease outside clinics [24]. For CDG, this approach successfully reported the experiences of CDG parents, identifying major healthcare and educational needs [25]. This community-centric approach also allowed us to detect changes in the impact of clinical manifestations over time. Specifically, infections were shown to be burdensome in infancy but not in adulthood. Contrastingly, skeletal manifestations (kyphosis/scoliosis and osteopenia) did not pose a problem until later in life. Even though there are reports of these time-dependent clinical occurrences [26, 27], there are no reports of their burden or impact on QoL for most clinical manifestations. Some pioneer attempts using patient-reported data were made to evaluate the impact of certain manifestations; however, they lacked the use of solid and validated questionnaires for that purpose [26, 28]. Our study responds to this gap by identifying the manifestations that families and experts prioritize across age ranges and by providing specific tools that can measure QoL related to those symptoms.

Our quantitative results highlight that both stakeholder groups (families and professionals) rated neurological signs as the most impactful across all age ranges, particularly hypotonia, developmental delay, ataxia, dysarthria, and intellectual disability. This was corroborated by the qualitative interviews since these are manifestations that impact all domains of QoL (physical, social, and mental functioning as well as the capacity to perform daily living activities) throughout the patients’ lives. Of note, these are also some of the most frequent clinical signs in PMM2-CDG patients [13]. Other neurological occurrences were also prioritized with lower impact scores, namely seizures and stroke-like episodes. These are mainly rare clinical events reported to happen in 13% and 7% of patients, respectively, but have been described as some of the most QoL-impacting issues in PMM2-CDG [13, 29, 30]. Surprisingly, much more pronounced impact scores are suggested by clinicians compared to families concerning these neurological manifestations. Considering the low frequency of these clinical signs, it is probable that the impact of these manifestations is underrated due to clinical representation bias. In fact, only 9/23 (39%) of families reported any kind of QoL impact (from mild to extreme) derived from stroke-like episodes at some point of the patient’s lives. Even though this percentage is still higher than the reported frequency, it might explain the low impact score from families since most of them rated stroke-like episodes as having no impact on their lives (IS = 1). Nevertheless, family-derived qualitative data indicates the physical, psychological, and mental burden attributed to these manifestations. Contrastingly, none of the seven family members interviewed were able to describe how peripheral neuropathy impacts their day-to-day life. Even though peripheral neuropathy was present in some of their clinical reports, the real and physical consequences (e.g., pain to touch, numbness, altered sensations) were not perceived by the interviewed families, which might explain the differences of impact perception compared to medical professionals. These observations show the importance of complementing patient-reported data with medical knowledge and experience. Given the low number of interviews performed, further studies should secure bigger patient cohorts of worldwide and differently aged patients and representation of the full clinical spectrum and severity of PMM2-CDG to further understand the burden of the disease accurately.

Other system-related manifestations were prioritized concordantly by families and professionals, namely ophthalmologic manifestations, infections, overheating episodes, behavioral problems, kyphosis/scoliosis, and osteopenia. On the contrary, families and doctors rated dysphagia and sex development deficiencies differently. Since dysphagia is often a consequence of hypotonia, while doctors have this knowledge, families might not have had the opportunity to become familiar about the difference between “dysphagia” and “difficulty swallowing due to muscle weakness (hypotonia)”. In fact, families rated hypotonia with high impact scores. A similar issue arises considering that food allergies were included as a QoL-impacting manifestation. Even though food allergies have been pointed out as having a negative impact in PMM2-CDG patients’ QoL [26], interviewed families did not experience this clinical issue. Moreover, the medical committee pinpointed that food allergies are extremely rare in PMM2-CDG but food intolerances are rather common. This inconsistency raises the possibility that families consider food “allergy” and “intolerance” interchangeable terms. Therefore, efforts should be taken to improve the communication between the medical teams and families, raise health literacy levels and contribute to the proper disease understanding and management. An action that could be taken in the future to help manage and minimise these differences encompasses creating and distributing glossaries explaining medical and difficult terms in lay-language to empower families to participate confidently. This methodology has proven helpful and effective in other people-centric studies [26, 31].

Some general patient-reported clinical assessment tools have already been used in clinics for PMM2-CDG, particularly the Goal Attainment Scale and the patient-centred measures from Patient-Reported Outcomes Measurement Information System (PROMIS) [32, 33]. However, none of these reflect the most impactful conditions presented by the patients. Our methodology answered this gap and allowed us to tailor our search for adequate PRO tools for most of the included disease manifestations. Importantly, 94.2% of the articles reporting the use of the 80 QoL tools identified used them as self-reports. This is normally considered as the best practice since it does not require interpretation by a proxy.[34] However, most included articles also reported PROMs use in mono-organ and non-neurologic diseases, mostly allowing the use of self-reporting. In the case of PMM2-CDG, the cognitive and motor impairment, as well as communication difficulties due to dyspraxia will restrain most patients to self-report how they feel and function. Proxy assessments—a proxy responding to a QoL tool aimed for self-reporting as they believe the patient would rate the items)—need to be put into place as a solution, as performed for other debilitating diseases [19, 35]. Typically, proxy reports tend to overestimate the QoL impact compared to self-rating, but it might depend on several factors (e.g., QoL domain, disease severity or difficulty of carer’s tasks). Nonetheless, in several instances, proxy-reports were found to correlate with self- assessments. Besides, we believe that the over or underestimation of QoL from proxies can be systematic and therefore, changes across time and following an intervention should be captured. Measuring clinical severity alongside proxy-reports might be a way to ensure their reliability [33]. However, we cannot discard that a minority of PMM2-CDG patients might be able to express themselves and provide QoL self-ratings. Different rating methods and creative tools should be available to ensure their inclusion.

We also aimed to objectively analyze the psychometric quality of the questionnaires, since they will be the basis for the development of a future PMM2-CDG QoL questionnaire. Our results showed that some psychometric properties are, in general, objectively calculated. Even so, for Construct Validity most articles did not explicitly present the hypotheses. This is a common and potential risk, as the instrument may not represent the intended construct [20]. On the contrary, most instruments showed a positive Content Validity rating, considered the most important psychometric property of a tool [36]. Since health-related questionnaires are essential to assess the impact of a disease or treatment, Agreement should be accessed in validation articles to define the absolute measurement error, required for evaluative purposes to distinguish clinically meaningful changes. However, properties such as Agreement, Floor and Ceiling effect, Responsiveness, Interpretability and Criterion Validity are rarely reported. Thus, future validations should include the assessment of these psychometric dimensions.

The recent advances in drug development programs and increase in clinical research for PMM2-CDG urge for a disease-appropriate and responsive HrQoL measure. This study is a step towards the development of this PMM2-CDG QoL questionnaire assuring the engagement and participation of families and doctors since its inception. Following efforts should adopt the same methodology complementing clinically important factors (doctors’ views) with aspects that make life worth living (patients’ and families’ views). Looking forward, item selection from the gathered questionnaires following standard development procedures and item reduction should follow a programmed decision system including all stakeholders and resorting to nominal groups and cognitive interviews.

## Limitations of this study

There were several limitations to this study. This is a pilot study that shows the potential of a community-centric methodology in PROMs development. Nevertheless, our results should be interpreted cautiously given the small sample participating in the impact survey and interviews. Even though our study aimed to include families representing different severities as well as different age ranges, the low number of families participating in the impact survey and the interviews are not representative of the full spectrum of PMM2-CDG clinical presentation, severity, and heterogeneity. As an example, we reached very limited representation of families of adult patients (n = 4 on the impact survey and n = 1 in the qualitative interviews). Next efforts should be taken to increase patient representation and capture the huge variability of clinical presentation of PMM2-CDG. Additionally, both in the questionnaire and interview, data on phenotypic severity should be collected to allow the stratification of the patient population according to disease severity and investigate if and how that affects HrQoL tool identification and, consequently PROMs development and/or administration.

We queried the PubMed database and no other sources because the project is led by a non-profit organisation without external funding. Non-English articles and articles using translated versions of the questionnaires were not included for practical reasons resulting in limited negative evidence. Although we are aware that we did not include all available instruments for the symptoms assessed, our methodology answered the main goal of this study—to identify the main questionnaires used across the impactful signs and symptoms. In our QoL tool quality analysis, the evaluation was centred on the criteria developed by Terwee et al., (2007), which are primarily opinion-based, and for which there is no empirical evidence to support explicit quality criteria in this field. However, it allowed us to establish a method of quality comparison between instruments. Also, there are some measurement properties that, despite being identified, are not assessed through the predefined criteria. Hence, there may be a need to “refine” or adapt these guidelines so that future comparative questionnaire analysis could be more accurate. Furthermore, the quality ratings do not consider a systematic review of validation studies associated with each questionnaire and depend on the availability of information on original development and validation articles. Finally, we cannot conclude that questionnaires with the highest number of positive ratings are necessarily the best ones, since some validation properties are more critical than others, depending on the aim of the questionnaire (e.g., discriminative questionnaires require a high level of reliability to be able to distinguish between people, while evaluative questionnaires require a high level of agreement to be able to measure essential changes). However, although important, this limitation did not interfere with the purpose of our analysis.

## Conclusions

Accurately measuring HrQoL using a PMM2-CDG-specific QoL questionnaire including the most concerning domains/symptoms from both the family and medical perspectives will benefit therapy development and approval but also for establishing the natural history of the disease in terms of QoL. In turn, it will require and benefit from the combined efforts from all stakeholders, particularly families, researchers, clinicians, and pharma representatives as shown in this study. As for other rare diseases, creative and new approaches for the development of such scales need to be applied, particularly given the clinical heterogeneity of PMM2-CDG throughout time and between patients. This study provides a solution for this matter particularly by surveying the patient and medical community about the most impactful symptoms through the lifespan of a patient providing a list of adequate tools/items for the development of a new specific scale.

## Supplementary Information

**Additional file 1**. Supplementary information of the methodology and results of the study: additional information concerning the semi-structured interviews guide (Table 1), the demographics of interviewed patients (Table 2), search keywords (Table 3) and the list of references of the included QoL tools (Table 4).

**Additional file 2**. Data collection sheet of semi-structured interviews and respective results.

**Additional file 3**. Quality assessment of included questionnaires.

## Data Availability

All data generated or analysed during this study are included in this published article [and its Additional files].

## Abbreviations

COA: Clinical Outcomes Assessment
CDG: Congenital Disorder(s) of Glycosylation
(Hr)QoL: (Health-related) Quality of Life
PRO(M): Patient-Reported Outcome (Measure)
ObsROM: Observer-Reported Outcomes Measure
