# Supplementary material for: Patient reported outcomes for phosphomannomutase 2 congenital disorder of glycosylation (PMM2-CDG): listening to what matters for the patients and health professionals
Source: Orphanet J Rare Dis. 2022 Oct 29;17:398. doi: 10.1186/s13023-022-02551-y (PMC9618201; doi:10.1186/s13023-022-02551-y)
Supplement: Supplementary file 1 — Additional file 1. Supplementary information of the methodology and results of the study: additional information concerning the semi-structured interviews guide (Table 1), the demographics of interviewed patients (Table 2), search keywords (Table 3) and the list of references of the included QoL tools (Table 4). [file 13023_2022_2551_MOESM1_ESM.docx]

**Additional File 1**

Supplementary information of the methodology and results of the study

**Table of Contents**

| Table 1 - Semi-structured interview guide. | 2 |
| --- | --- |
| Table 2 - Demographics of the 7 PMM2-CDG patients included in proxy qualitative interviews. | 3 |
| Table 3 - Groups of keywords used to build the search query. | 4 |
| Table 4 - List of references for the included symptom/disease-specific quality of life tools. | 5 |

Table 1 – Semi-structured interview guide.

| **Section** | **Step** | **Description** |
| --- | --- | --- |
| Introduction | 1 | Interviewer introduction |
|  | 2 | Project summary. |
|  | 3 | Research objectives and aim of interview. Explanation that interviewee may interrupt whenever there is need to clarification. |
|  | 4 | Call settings (confidential, not recorded but notes will be taken, open-end questions, prediction of 60 minutes) |
|  | 5 | Importance of the collection of this type of data. |
| CDG symptoms impact | 6 | What is your (parents of children and adults with CDG) experience with *[SIGN/SYMPTOM]*? |
|  | 7 | What are the perceived consequences of the *[SIGN/MANIFESTATION]* on your child or on the family dynamics? |
|  |  | (OR) What is the real meaning or impact of *[SIGN/MANIFESTATION]* on your child or your family daily life? |
|  |  | (OR) In what way does having *[SIGN/MANIFESTATION]* impacts on your quality of life? |
|  |  |  |
|  |  | List of SIGNS/MANIFESTATIONS on the data collection sheet (Supplementary File 2) |
| Closing interview | 8 | Acknowledgment |
|  | 9 | Availability of results |
|  | 10 | Farewell |

Table 2 - Demographics of the 7 PMM2-CDG patients included in proxy qualitative interviews.

| Patient | Country | Age (years old) |
| --- | --- | --- |
| 1 | Portugal | 40 |
| 2 | Portugal | 5 |
| 3 | Switzerland | 7 |
| 4 | Portugal | 6 |
| 5 | Netherlands | 25 |
| 6 | United Kingdom | 10 |
| 7 | Spain | 10 |

Table 3 - Groups of keywords used to build the search query. Keywords within the group were connected using the boolean operator “OR” while the different groups were connected using the operator “AND”. For some signs and symptoms, the keywords AND (“metabolic” OR “rare disease”) were added to the combination to provide more specific results.

| **Signs and symptoms keywords** | **(AND)**  **Patient reported outcomes keywords** | **(AND)**  **Quality of Life keywords** |
| --- | --- | --- |
| ("Behavioral problems" OR "Aggressive behaviour") | (“patient reported outcomes”  OR “PRO”  OR “patient reported outcome measure”  OR “PROM”  OR “observer reported outcomes”  OR “observer reported outcome measure”  OR “patient centered outcomes”  OR “patient centred outcomes”  OR “proxy reported outcomes”  OR “survey”  OR “questionnaire”) | (“quality of life”  OR “health related quality of life”  OR“HRQOL”  OR “disease-specific quality of life”) |
| ("Developmental delay" OR "Failure to thrive" OR "Gross motor delay" OR "Fine motor delay" OR "psychological functioning" "Social development" OR "Social functioning" OR "social isolation" OR "emotional development" OR "emotional functioning" OR "Intellectual disability" OR "Learning disability" OR "Mental retardation")* |  |  |
| (Dysphagia OR "Tube feeding" OR "Enteral nutrition" OR Malabsorption OR Enteropathy OR Diarrhea OR vomiting)* |  |  |
| (Dysarthria OR "Speech delay" OR "Language delay") |  |  |
| Hypotonia |  |  |
| Infections* |  |  |
| (Kyphosis OR Scoliosis)* |  |  |
| (Strabismus OR "Retinitis Pigmentosa" OR Esotropia OR nystagmus OR myopia) |  |  |
| Ataxia |  |  |
| Osteopenia* |  |  |
| "Peripheral neuropathy"* |  |  |
| (Seizures OR epilepsy OR convulsions)* |  |  |
| Stroke-like episodes |  |  |
| Coagulopathy* |  |  |
| ("Sleep disturbances" OR insomnia)* |  |  |
| "Food allergy"* |  |  |
| ("hypergonadotropic hypogonadism" OR "delayed puberty" OR "incomplete puberty" OR "absent puberty" OR amenorrhea) |  |  |
| (hepatomegaly OR "fatty liver" OR steatosis OR Cirrhosis OR "Liver failure")* |  |  |

Table 4 - List of references for the included symptom/disease-specific quality of life tools.

| **Symptom/disease-specific quality of life tool** | **References** |
| --- | --- |
| 10-item Neuro-Ophthalmic Supplement (NOS) to the NEI-VFQ-25 | - Ihl T, Kadas EM, Oberwahrenbrock T, Endres M, Klockgether T, Schroeter J, et al. Investigation of Visual System Involvement in Spinocerebellar Ataxia Type 14. Cerebellum. 2020;19(4):469-482. - Kedar S, Ghate D, Murray EL, Corbett JJ, Subramony SH. Vision related quality of life in spinocerebellar ataxia. J Neurol Sci. 2015;358(1-2):404-8. |
| 8-question QoL live interview developed by Kothari M et al., (2009) | - Kothari M, Balankhe S, Gawade R, Toshnival S. Comparison of psychosocial and emotional consequences of childhood strabismus on the families from rural and urban India. Indian J Ophthalmol. 2009;57(4):285-8. |
| Adult Strabismus Quality of Life Questionnaire - 11 item (AS-11) | - Gothwal VK, Bharani S, Kekunnaya R, Chhablani P, Sachdeva V, Pehere NK, Narasaiah A, et al. Measuring Health-Related Quality of Life in Strabismus: A Modification of the Adult Strabismus-20 (AS-20) Questionnaire Using Rasch Analysis. PLoS One. 2015;10(5):e0127064. doi: 10.1371/journal.pone.0127064. |
| Adult Strabismus Quality of Life Questionnaire (AS-20) | - Hatt SR, Leske DA, Iezzi R Jr, Holmes JM. Binocular Interference vs Diplopia in Patients With Epiretinal Membrane. JAMA Ophthalmol. 2020;138(11):1121-1127. - Wang JY, Leske DA, Hatt SR, Holmes JM. Diplopia after strabismus surgery for adults with nondiplopic childhood-onset strabismus. J AAPOS. 2019;23(6):313.e1-313.e5. - Sim PY, Cleland C, Dominic J, Jain S. Investigation of factors associated with the success of adult strabismus surgery from the patient's perspective. J AAPOS. 2018;22(4):266-271.e3. - Khanna CL, Leske DA, Holmes JM. Factors Associated With Health-Related Quality of Life in Medically and Surgically Treated Patients With Glaucoma. JAMA Ophthalmol. 2018;136(4):348-355. - Sah SP, Sharma IP, Chaudhry M, Saikia M. Health-Related Quality of Life (HRQoL) in Young Adults with Strabismus in India. J Clin Diagn Res. 2017;11(2):NC01-NC04. - Leske DA, Hatt SR, Liebermann L, Holmes JM. Lookup Tables Versus Stacked Rasch Analysis in Comparing Pre- and Post intervention Adult Strabismus-20 Data. Transl Vis Sci Technol. 2016;5(1):11. - Tandon AK, Velez FG, Isenberg SJ, Demer JL, Pineles SL. Binocular inhibition in strabismic patients is associated with diminished quality of life. J AAPOS. 2014;18(5):423-6. - Alam D, Khan AA, Bani SA, Sharma R, Amitava AK. Gain beyond cosmesis: demonstration of psychosocial and functional gains following successful strabismus surgery using the adult strabismus questionnaire adult strabismus 20. Indian J Ophthalmol. 2014;62(7):799-803. - Saunte JP, Holmes JM. Sustained improvement of reading symptoms following botulinum toxin A injection for convergence insufficiency. Strabismus. 2014;22(3):95-9. - Sim B, Yap GH, Chia A. Functional and psychosocial impact of strabismus on Singaporean children. J AAPOS. 2014;18(2):178-82. - Liebermann L, Hatt SR, Leske DA, Holmes JM. Improvement in specific function-related quality-of-life concerns after strabismus surgery in nondiplopic adults. J AAPOS. 2014;18(2):105-9. - Hatt SR, Leske DA, Liebermann L, Holmes JM. Successful treatment of diplopia with prism improves health-related quality of life. Am J Ophthalmol. 2014;157(6):1209-13. - Glasman P, Cheeseman R, Wong V, Young J, Durnian JM. Improvement in patients' quality-of-life following strabismus surgery: evaluation of postoperative outcomes using the Adult Strabismus 20 (AS-20) score. Eye (Lond). 2013;27(11):1249-53. - Holmes JM, Liebermann L, Hatt SR, Smith SJ, Leske DA. Quantifying diplopia with a questionnaire. Ophthalmology. 2013;120(7):1492-6. - ​​Leske DA, Hatt SR, Liebermann L, Holmes JM. Evaluation of the Adult Strabismus-20 (AS-20) questionnaire using Rasch analysis. Invest Ophthalmol Vis Sci. 2012;53(6):2630-9. - Hancox J, Sharma S, MacKenzie K, Adams G. The effect on quality of life of long-term botulinum toxin A injections to maintain ocular alignment in adult patients with strabismus. Br J Ophthalmol. 2012;96(6):838-40. - Hatt SR, Leske DA, Liebermann L, Holmes JM. Changes in health-related quality of life 1 year following strabismus surgery. Am J Ophthalmol. 2012;153(4):614-9. - Durnian JM, Owen ME, Baddon AC, Noonan CP, Marsh IB. The psychosocial effects of strabismus: effect of patient demographics on the AS-20 score. J AAPOS. 2010;14(6):469-71. - Hatt SR, Leske DA, Holmes JM. Responsiveness of health-related quality-of-life questionnaires in adults undergoing Strabismus surgery. Ophthalmology. 2010;117(12):2322-2328.e1. - Leske DA, Hatt SR, Holmes JM. Test-retest reliability of health-related quality-of-life questionnaires in adults with strabismus. Am J Ophthalmol. 2010;149(4):672-6. - Durnian JM, Owen ME, Marsh IB. The psychosocial aspects of strabismus: correlation between the AS-20 and DAS59 quality-of-life questionnaires. J AAPOS. 2009;13(5):477-80. - Hatt SR, Leske DA, Bradley EA, Cole SR, Holmes JM. Comparison of quality-of-life instruments in adults with strabismus. Am J Ophthalmol. 2009;148(4):558-62 - Hatt SR, Leske DA, Bradley EA, Cole SR, Holmes JM. Development of a quality-of-life questionnaire for adults with strabismus. Ophthalmology. 2009;116(1):139-144.e5. |
| Amblyopia and Strabismus Questionnaire (A&SQ) | - Chen Y, Chen X, Chen J, Zheng J, Xu J, Yu X. Longitudinal Impact on Quality of Life for School-aged Children with Amblyopia Treatment: Perspective from Children. Curr Eye Res. 2016;41(2):208-14. - Tandon AK, Velez FG, Isenberg SJ, Demer JL, Pineles SL. Binocular inhibition in strabismic patients is associated with diminished quality of life. J AAPOS. 2014;18(5):423-6. - Bujak MC, Leung AK, Kisilevsky M, Margolin E. Monovision correction for small-angle diplopia. Am J Ophthalmol. 2012;154(3):586-592.e2. - van de Graaf ES, van der Sterre GW, van Kempen-du Saar H, Simonsz B, Looman CW, Simonsz HJ. Amblyopia and Strabismus Questionnaire (A&SQ): clinical validation in a historic cohort. Graefes Arch Clin Exp Ophthalmol. 2007;245(11):1589-95. - Felius J, Beauchamp GR, Stager DR Sr, Van De Graaf ES, Simonsz HJ. The Amblyopia and Strabismus Questionnaire: English translation, validation, and subscales. Am J Ophthalmol. 2007;143(2):305-310. - van de Graaf ES, van der Sterre GW, Polling JR, van Kempen H, Simonsz B, Simonsz HJ. Amblyopia & Strabismus Questionnaire: design and initial validation. Strabismus. 2004;12(3):181-93. |
| Canadian Haemophilia Outcomes-Kids’ Life Assessment Tool (CHO-KLAT) | - Crivianu-Gaita V, Rivard GE, Carcao M, Teitel J, St-Louis J, Blanchette V, et al. Pilot study of once-a-day prophylaxis for youth and young adults with severe haemophilia A. Multicenter Study Haemophilia. 2016;22(5):e401-5. |
| Celiac disease-specific DUX questionnaire (CDDUX) | - van Doorn RK, Winkler LMF, Zwinderman KH, Mearin ML, Koopman HM. CDDUX: a disease-specific health-related quality-of-life questionnaire for children with celiac disease. J. Pediatr. Gastroenterol. Nutr.. [2008;47(2):147-152](https://journals.lww.com/jpgn/toc/2008/08000) |
| Chronic Liver Disease Questionnaire (CLDQ) | - Wunsch E, Koziarska D, Milkiewicz M, Naprawa G, Nowacki P, Hartleb M, et al. In patients with liver cirrhosis, proinflammatory interleukins correlate with health-related quality of life irrespective of minimal hepatic encephalopathy. Eur J Gastroenterol Hepatol. 2013;25(12):1402-7. - Wunsch E, Naprawa G, Koziarska D, Milkiewicz M, Nowacki P, Milkiewicz P. Serum natremia affects health-related quality of life in patients with liver cirrhosis: a prospective, single centre study. Ann Hepatol. 2013;12(3):448-55. - Parkash O, Iqbal R, Jafri F, Azam I, Jafri W. Frequency of poor quality of life and predictors of health related quality of life in cirrhosis at a tertiary care hospital Pakistan. BMC Res Notes. 2012;5:446. - Sanyal A, Younossi ZM, Bass NM, Mullen KD, Poordad F, Brown RS, et al. Randomised clinical trial: rifaximin improves health-related quality of life in cirrhotic patients with hepatic encephalopathy - a double-blind placebo-controlled study. Aliment Pharmacol Ther. 2011;34(8):853-61 - Younossi ZM, Kiwi ML, Boparai N, Price LL, Guyatt G. Cholestatic liver diseases and health-related quality of life. Am J Gastroenterol. 2000;95(2):497-502. |
| CLDQ for NAFLD NASH (CLDQ NAFLD-NASH) | - O'Hara J, Finnegan A, Dhillon H, Ruiz-Casas L, Pedra G, Franks B, et al. Cost of non-alcoholic steatohepatitis in Europe and the USA: The GAIN study. JHEP Rep. 2020;2(5):100142. - Younossi ZM, Stepanova M, Younossi I, Racila A. Validation of Chronic Liver Disease Questionnaire for Nonalcoholic Steatohepatitis in Patients With Biopsy-Proven Nonalcoholic Steatohepatitis. Clin Gastroenterol Hepatol. 2019;17(10):2093-2100.e3. |
| College of Optometrists in Vision Development Quality of Life questionnaire (COVD QOL) | - Hussey ES. Remote treatment of intermittent central suppression improves quality-of-life measures. Optometry. 2012;83(1):19-26. |
| Cushing QoL questionnaire (CushQoL) | - Sarkis P, Rabilloud M, Lifante JC, Siamand A, Jouanneau E, Gay E, et al. Bilateral adrenalectomy in Cushing's disease: Altered long-term quality of life compared to other treatment options. Ann Endocrinol (Paris). 2019;80(1):32-37. - Osswald A, Deutschbein T, Berr CM, Plomer E, Mickisch A, Ritzel K, et al. Surviving ectopic Cushing's syndrome: quality of life, cardiovascular and metabolic outcomes in comparison to Cushing's disease during long-term follow-up. Eur J Endocrinol. 2018;179(2):109-116. |
| Deglutition Handicap Index (DHI) | - Woisard V, Lepage B. The "Deglutition Handicap Index" a self-administrated dysphagia-specific quality of life questionnaire: temporal reliability. Rev Laryngol Otol Rhinol (Bord). 2010;131(1):19-22 |
| Disease specific questionnaire based on the study of the Joint LASIK Study Task Force (Schallhorn et al., 2016) | - Schallhorn SC, Venter JA, Teenan D, Hannan SJ, Hettinger KA, Pelouskova M, et al. Patient-reported outcomes 5 years after laser in situ keratomileusis. J Cataract Refract Surg. 2016;42(6):879-89. |
| Dysarthria Impact Profile (DIP) | - Walshe M, Peach RK, Miller N. Dysarthria impact profile: development of a scale to measure psychosocial effects. Int J Lang Commun Disord. 2009;44(5):693-715. |
| Dysphagia Handicap Index (DHI) | - Jaffar S, Devadas M. Characterization of Self-Reported Dysphagia and Impact on Weight Outcomes After Laparoscopic Sleeve Gastrectomy. Obes Surg. 2018;28(10):3177-3185. |
| Early-Onset Scoliosis Questionnaire (EOSQ-24) | - Johnston CE, Tran DP, McClung A. Functional and Radiographic Outcomes Following Growth-Sparing Management of Early-Onset Scoliosis. J Bone Joint Surg Am. 2017;99(12):1036-1042. |
| Eating Assessment Tool (EAT-10) | - Belafsky PC, Mouadeb DA, Rees CJ, Pryor JC, Postma GN, Allen J, et al. Validity and reliability of the Eating Assessment Tool (EAT-10). Ann Otol Rhinol Laryngol. 2008;117(12):919-24. |
| EORTC QLQ - OES18 - Oesophageal Module 18 | - Larsson H, Norder GE, Tegtmeyer B, Ruth M, Bergquist H, Bove M. Grade of eosinophilia versus symptoms in patients with dysphagia and esophageal eosinophilia. Dis Esophagus. 2016;29(8):971-976. - Donohoe CL, Healy LA, Fanning M, Doyle SL, Hugh AM, Moore J, et al. Impact of supplemental home enteral feeding post esophagectomy on nutrition, body composition, quality of life, and patient satisfaction. Dis Esophagus. 2017;30(9):1-9. |
| EORTC QLQ-GINET21 | - Lewis AR, Wang X, Magdalani L, D'Arienzo P, Bashir C, Mansoor W, et al. Health-related quality of life, anxiety, depression and impulsivity in patients with advanced gastroenteropancreatic neuroendocrine tumours. World J Gastroenterol. 2018;24(6):671-679. |
| Epilepsy & Learning Disabilities Quality of Life Questionnaire (ELDQOL) | - Brunklaus A, Dorris L, Zuberi SM. Comorbidities and predictors of health-related quality of life in Dravet syndrome. Epilepsia. 2011;52(8):1476-82. |
| Feeding / Swallowing - Impact Survey (FS-IS) | - Kokje VBC, Mermod M, Bertinazzi M, Sandu K. A new dimension of success in the management of airway disease in children with neurological deficit. Int J Pediatr Otorhinolaryngol. 2020;139:110483 |
| Food Allergy Quality of Life Questionnaire - Parent Form (FAQLQ-PF) | - DunnGalvin A, Koman E, Raver E, Frome H, Adams M, Keena A, et al. An Examination of the Food Allergy Quality of Life Questionnaire Performance in a Countrywide American Sample of Children: Cross-Cultural Differences in Age and Impact in the United States and Europe. J Allergy Clin Immunol Pract. 2017;5(2):363-368.e2 |
| Gastroesophageal Reflux Disease-Health Related Quality of Life (GERD-HRQL) | - Louie BE, Smith CD, Smith CC, Bell RCW, Gillian GK, Mandel JS, et al. Objective Evidence of Reflux Control After Magnetic Sphincter Augmentation: One Year Results From a Post Approval Study. Ann Surg. 2019;270(2):302-308. - Franceschelli S, Gatta DMP, Pesce M, Ferrone A, Di Martino G, Di Nicola M, et al. Modulation of the oxidative plasmatic state in gastroesophageal reflux disease with the addition of rich water molecular hydrogen: A new biological vision. J Cell Mol Med. 2018;22(5):2750-2759. - Noar MD, Lotfi-Emran S. Sustained improvement in symptoms of GERD and antisecretory drug use: 4-year follow-up of the Stretta procedure. Gastrointest Endosc. 2007;65(3):367-72. - Pleskow D, Rothstein R, Lo S, Hawes R, Kozarek R, Haber G, et al. Endoscopic full-thickness plication for the treatment of GERD: 12-month follow-up for the North American open-label trial. Gastrointest Endosc. 2005;61(6):643-9 |
| GastroIntestinal Quality of Life Index (GIQLI) | - Lee WJ, Lee MH, Yu PJ, Wei JH, Chong K, Chen SC, et al. Gastro-intestinal Quality of Life After Metabolic Surgery for the Treatment of Type 2 Diabetes Mellitus. Obes Surg. 2015;25(8):1371-9. - Yu PJ, Tsou JJ, Lee WJ, Lee KT, Lee YC. Impairment of gastrointestinal quality of life in severely obese patients. World J Gastroenterol. 2014;20(22):7027-33 - McLeod RS, Taylor BR, O'Connor BI, Greenberg GR, Jeejeebhoy KN, Royall D, et al. Quality of life, nutritional status, and gastrointestinal hormone profile following the Whipple procedure. Am J Surg. 1995;169(1):179-85. |
| Haemophilia-specific health- related quality of life questionnaire for adults (HAEMO-QoL-A) | - Stemberger M, Kallenbach F, Schmit E, McEneny-King A, Germini F, Yeung CHT, et al. Impact of Adopting Population Pharmacokinetics for Tailoring Prophylaxis in Haemophilia A Patients: A Historically Controlled Observational Study. Thromb Haemost. 2019;119(3):368-376. - Oldenburg J, Mahlangu JN, Bujan W, Trask P, Callaghan MU, Young G, et al. The effect of emicizumab prophylaxis on health-related outcomes in persons with haemophilia A with inhibitors: HAVEN 1 Study. Haemophilia. 2019;25(1):33-44. - Dekoven M, Wisniewski T, Petrilla A, Holot N, Lee WC, Cooper DL, et al. Health-related quality of life in haemophilia patients with inhibitors and their caregivers. Haemophilia. 2013;19(2):287-93. |
| Haemophilia-specific health-related quality of life questionnaire - short form (HAEMO-QoL-SF) | - Oldenburg J, Mahlangu JN, Bujan W, Trask P, Callaghan MU, Young G, et al. The effect of emicizumab prophylaxis on health-related outcomes in persons with haemophilia A with inhibitors: HAVEN 1 Study. Haemophilia. 2019;25(1):33-44. |
| Home parenteral nutrition-quality of life (HPN-QOL) | - Baxter JP, Fayers PM, Bozzetti F, Kelly D, Joly F, Wanten G, et al. An international study of the quality of life of adult patients treated with home parenteral nutrition. Clin Nutr. 2019;38(4):1788-1796. |
| HRQOL questionnaire developed by Chen et al., 2015 | - Chen Y, Chen X, Chen J, Zheng J, Xu J, Yu X. Longitudinal Impact on Quality of Life for School-aged Children with Amblyopia Treatment: Perspective from Children. Curr Eye Res. 2016;41(2):208-14. |
| IBDQ (no version specified) | - Margalit M, Israeli E, Shibolet O, Zigmond E, Klein A, Hemed N, et al. A double-blind clinical trial for treatment of Crohn's disease by oral administration of Alequel, a mixture of autologous colon-extracted proteins: a patient-tailored approach. Am J Gastroenterol. 2006;101(3):561-8 - Eser A, Colombel JF, Rutgeerts P, Vermeire S, Vogelsang H, Braddock M, et al. Safety and Efficacy of an Oral Inhibitor of the Purinergic Receptor P2X7 in Adult Patients with Moderately to Severely Active Crohn's Disease: A Randomized Placebo-controlled, Double-blind, Phase IIa Study. Inflamm Bowel Dis. 2015;21(10):2247-53 |
| IBDQ-32 | - Hanauer S, Sandborn WJ, Colombel JF, Vermeire S, Petersson J, Kligys K et al. Rapid Changes in Laboratory Parameters and Early Response to Adalimumab: A Pooled Analysis From Patients With Ulcerative Colitis in Two Clinical Trials. J Crohns Colitis. 2019;13(9):1227-1233. - Wiestler M, Kockelmann F, Kück M, Kerling A, Tegtbur U, Manns MP, et al. Quality of Life Is Associated With Wearable-Based Physical Activity in Patients With Inflammatory Bowel Disease: A Prospective, Observational Study. Clin Transl Gastroenterol. 2019;10(11):e00094. - Bjarnason I, Sission G, Hayee B. A randomised, double-blind, placebo-controlled trial of a multi-strain probiotic in patients with asymptomatic ulcerative colitis and Crohn's disease. Inflammopharmacology. 2019;27(3):465-473. - Eliadou E, Kini G, Huang J, Champion A, Inns SJ. Intravenous Iron Replacement Improves Quality of Life in Hypoferritinemic Inflammatory Bowel Disease Patients with and without Anemia. Dig Dis. 2017;35(5):444-448. - Artom M, Czuber-Dochan W, Sturt J, Murrells T, Norton C. The contribution of clinical and psychosocial factors to fatigue in 182 patients with inflammatory bowel disease: a cross-sectional study. Aliment Pharmacol Ther. 2017;45(3):403-416. - Miklavcic JJ, Shoemaker GK, Schnabl KL, Larsen BMK, Thomson ABR, Mazurak VC, et al. Ganglioside Intake Increases Plasma Ganglioside Content in Human Participants. JPEN J Parenter Enteral Nutr. 2017;41(4):657-666. - Brandse JF, Vos LM, Jansen J, Schakel T, Ponsioen CI, van den Brink GR, et al. Serum Concentration of Anti-TNF Antibodies, Adverse Effects and Quality of Life in Patients with Inflammatory Bowel Disease in Remission on Maintenance Treatment. J Crohns Colitis. 2015;9(11):973-81. - Gerbarg PL, Jacob VE, Stevens L, Bosworth BP, Chabouni F, DeFilippis EM, et al. The Effect of Breathing, Movement, and Meditation on Psychological and Physical Symptoms and Inflammatory Biomarkers in Inflammatory Bowel Disease: A Randomized Controlled Trial. Inflamm Bowel Dis. 2015;21(12):2886-96. - Kuo B, Bhasin M, Jacquart J, Scult MA, Slipp L, Riklin EI, et al. Genomic and clinical effects associated with a relaxation response mind-body intervention in patients with irritable bowel syndrome and inflammatory bowel disease. PLoS One. 2015;10(4):e0123861. - Yokoyama Y, Watanabe K, Ito H, Nishishita M, Sawada K, Okuyama Y, et al. Factors associated with treatment outcome, and long-term prognosis of patients with ulcerative colitis undergoing selective depletion of myeloid lineage leucocytes: a prospective multicenter study. Cytotherapy. 2015;17(5):680-8. - Brotherton CS, Taylor AG, Bourguignon C, Anderson JG. A high-fiber diet may improve bowel function and health-related quality of life in patients with Crohn disease. Gastroenterol Nurs. 2014;37(3):206-16 - Jedel S, Hoffman A, Merriman P, Swanson B, Voigt R, Rajan KB, et al. A randomized controlled trial of mindfulness-based stress reduction to prevent flare-up in patients with inactive ulcerative colitis. Digestion. 2014;89(2):142-55. - Vogelaar L, van't Spijker A, Timman R, van Tilburg AJ, Bac D, Vogelaar T, et al. Fatigue management in patients with IBD: a randomised controlled trial. Gut. 2014;63(6):911-8. - Dryden GW, Lam A, Beatty K, Qazzaz HH, McClain CJ. A pilot study to evaluate the safety and efficacy of an oral dose of (-)-epigallocatechin-3-gallate-rich polyphenon E in patients with mild to moderate ulcerative colitis. Inflamm Bowel Dis. 2013;19(9):1904-12. - Surti B, Spiegel B, Ippoliti A, Vasiliauskas EA, Simpson P, Shih DQ, et al. Assessing health status in inflammatory bowel disease using a novel single-item numeric rating scale. Dig Dis Sci. 2013;58(5):1313-21. - Bassaganya-Riera J, Hontecillas R, Horne WT, Sandridge M, Herfarth HH, Bloomfeld R, et al. Conjugated linoleic acid modulates immune responses in patients with mild to moderately active Crohn's disease. Clin Nutr. 2012;31(5):721-7. - Graff LA, Vincent N, Walker JR, Clara I, Carr R, Ediger J, et al. A population-based study of fatigue and sleep difficulties in inflammatory bowel disease. Inflamm Bowel Dis. 2011;17(9):1882-9. - Wiese DM, Lashner BA, Lerner E, DeMichele SJ, Seidner DL. The effects of an oral supplement enriched with fish oil, prebiotics, and antioxidants on nutrition status in Crohn's disease patients. Nutr Clin Pract. 2011;26(4):463-73. - Schmidt C, Häuser W, Giese T, Stallmach A. Irritable pouch syndrome is associated with depressiveness and can be differentiated from pouchitis by quantification of mucosal levels of proinflammatory gene transcripts. Inflamm Bowel Dis. 2007;13(12):1502-8. - Wells CW, Lewis S, Barton JR, Corbett S. Effects of changes in hemoglobin level on quality of life and cognitive function in inflammatory bowel disease patients. Inflamm Bowel Dis. 2006;12(2):123-30. - de Silva AD, Tsironi E, Feakins RM, Rampton DS. Efficacy and tolerability of oral iron therapy in inflammatory bowel disease: a prospective, comparative trial. Aliment Pharmacol Ther. 2005;22(11-12):1097-105. - Rutgeerts P, D'Haens G, Targan S, Vasiliauskas E, Hanauer SB, Present DH, et al. Efficacy and safety of retreatment with anti-tumor necrosis factor antibody (infliximab) to maintain remission in Crohn's disease. Gastroenterology. 1999;117(4):761-9. - Greenberg GR, Feagan BG, Martin F, Sutherland LR, Thomson AB, Williams CN, et al. Oral budesonide for active Crohn's disease. Canadian Inflammatory Bowel Disease Study Group. N Engl J Med. 1994;331(13):836-41. |
| IBDQ-36 | - Love JR, Irvine EJ, Fedorak RN. Quality of life in inflammatory bowel disease. J Clin Gastroenterol. 1992;14(1):15-9. |
| IBDQ-9 | - Sadeghi N, Mansoori A, Shayesteh A, Hashemi SJ. The effect of curcumin supplementation on clinical outcomes and inflammatory markers in patients with ulcerative colitis. Phytother Res. 2020;34(5):1123-1133. - Karimi S, Tabataba-Vakili S, Yari Z, Alborzi F, Hedayati M, Ebrahimi-Daryani N, et al. The effects of two vitamin D regimens on ulcerative colitis activity index, quality of life and oxidant/anti-oxidant status. Nutr J. 2019;18(1):16. - Nikkhah-Bodaghi M, Darabi Z, Agah S, Hekmatdoost A. The effects of Nigella sativa on quality of life, disease activity index, and some of inflammatory and oxidative stress factors in patients with ulcerative colitis. Phytother Res. 2019;33(4):1027-1032. - Abautret-Daly Á, Dempsey E, Riestra S, de Francisco-García R, Parra-Blanco A, Rodrigo L, et al. Association between psychological measures with inflammatory and disease-related markers of inflammatory bowel disease. Int J Psychiatry Clin Pract. 2017;21(3):221-230. - de Vries SAG, Tan CXW, Bouma G, Forouzanfar T, Brand HS, de Boer NK. Salivary Function and Oral Health Problems in Crohn's Disease Patients. Inflamm Bowel Dis. 2018;24(6):1361-1367. |
| Insomnia Severity Index (ISI) | - Sharifan P, Khoshakhlagh M, Khorasanchi Z, Darroudi S, Rezaie M, Safarian M, et al. Efficacy of low-fat milk and yogurt fortified with encapsulated vitamin D(3) on improvement in symptoms of insomnia and quality of life: Evidence from the SUVINA trial. Food Sci Nutr. 2020;8(8):4484-4490. |
| Intermittent Exotropia Questionnaire (IXTQ) | - Wang Y, Xu M, Yu H, Xu J, Hou F, Zhou J, et al. Health-related quality of life correlated with the clinical severity of intermittent exotropia in children. Eye (Lond). 2020;34(2):400-407. - Hatt SR, Leske DA, Liebermann L, Holmes JM. Symptoms in Children with Intermittent Exotropia and Their Impact on Health-Related Quality of Life. Strabismus. 2016;24(4):139-145. - Lim SB, Wong WL, Ho RC, Wong IB. Childhood intermittent exotropia from a different angle: does severity affect quality of life? Br J Ophthalmol. 2015;99(10):1405-11. - Sim B, Yap GH, Chia A. Functional and psychosocial impact of strabismus on Singaporean children. J AAPOS. 2014;18(2):178-82. - Hatt SR, Leske DA, Liebermann L, Mohney BG, Brodsky MC, Yamada T, et al. Associations between health-related quality of life and the decision to perform surgery for childhood intermittent exotropia. Ophthalmology. 2014;121(4):883-8. - Yamada T, Hatt SR, Leske DA, Holmes JM. Specific health-related quality of life concerns in children with intermittent exotropia. Strabismus. 2012;20(4):145-51. - Yamada T, Hatt SR, Leske DA, Holmes JM. Spectacle wear in children reduces parental health-related quality of life. J AAPOS. 2011;15(1):24-8. - Hatt SR, Leske DA, Holmes JM. Comparison of quality-of-life instruments in childhood intermittent exotropia. J AAPOS. 2010;14(3):221-6. - Hatt SR, Leske DA, Yamada T, Bradley EA, Cole SR, Holmes JM. Development and initial validation of quality-of-life questionnaires for intermittent exotropia. Ophthalmology. 2010;117(1):163-168.e1. |
| Irritable Bowel Syndrome Quality of Life (IBS-QOL) | - Panarese A, Pesce F, Porcelli P, Riezzo G, Iacovazzi PA, Leone CM, et al. Chronic functional constipation is strongly linked to vitamin D deficiency. World J Gastroenterol. 2019;25(14):1729-1740. - Harvie RM, Chisholm AW, Bisanz JE, Burton JP, Herbison P, Schultz K, et al. Long-term irritable bowel syndrome symptom control with reintroduction of selected FODMAPs. World J Gastroenterol. 2017;23(25):4632-4643. - Pedersen N, Ankersen DV, Felding M, Wachmann H, Végh Z, Molzen L, et al. Low-FODMAP diet reduces irritable bowel symptoms in patients with inflammatory bowel disease. World J Gastroenterol. 2017;23(18):3356-3366. - Choghakhori R, Abbasnezhad A, Hasanvand A, Amani R. Inflammatory cytokines and oxidative stress biomarkers in irritable bowel syndrome: Association with digestive symptoms and quality of life. Cytokine. 2017;93:34-43. - Choghakhori R, Abbasnezhad A, Amani R, Alipour M. Sex-Related Differences in Clinical Symptoms, Quality of Life, and Biochemical Factors in Irritable Bowel Syndrome. Dig Dis Sci. 2017;62(6):1550-1560. - Lyra A, Hillilä M, Huttunen T, Männikkö S, Taalikka M, Tennilä J, et al. Irritable bowel syndrome symptom severity improves equally with probiotic and placebo. World J Gastroenterol. 2016;22(48):10631-10642. - Kuo B, Bhasin M, Jacquart J, Scult MA, Slipp L, Riklin EI, et al. Genomic and clinical effects associated with a relaxation response mind-body intervention in patients with irritable bowel syndrome and inflammatory bowel disease. PLoS One. 2015;10(4):e0123861. - Böhn L, Störsrud S, Törnblom H, Bengtsson U, Simrén M. Self-reported food-related gastrointestinal symptoms in IBS are common and associated with more severe symptoms and reduced quality of life. Am J Gastroenterol. 2013;108(5):634-41. |
| Irritable Bowel Syndrome Quality of Life Questionnaire (IBSQoL) | - Böhn L, Störsrud S, Törnblom H, Bengtsson U, Simrén M. Self-reported food-related gastrointestinal symptoms in IBS are common and associated with more severe symptoms and reduced quality of life. Am J Gastroenterol. 2013;108(5):634-41. |
| Izumo scale for abdominal symptom-related QOL | - Fujishiro M, Kushiyama A, Yamazaki H, Kaneko S, Koketsu Y, Yamamotoya T, et al. Gastrointestinal symptom prevalence depends on disease duration and gastrointestinal region in type 2 diabetes mellitus. World J Gastroenterol. 2017;23(36):6694-6704. - Yoshioka T, Okimoto N, Okamoto K, Sakai A. A comparative study of the effects of daily minodronate and weekly alendronate on upper gastrointestinal symptoms, bone resorption, and back pain in postmenopausal osteoporosis patients. J Bone Miner Metab. 2013;31(2):153-60. - Kakuta E, Yamashita N, Katsube T, Kushiyama Y, Suetsugu H, Furuta K, et al. Abdominal Symptom-related QOL in Individuals Visiting an Outpatient Clinic and those Attending an Annual Health Check. Intern Med. 2011;50:1517-1522 |
| Kansas City Cardiomyopathy Questionnaire (KCCQ-12) | - Armstrong PW, Lam CSP, Anstrom KJ, Ezekowitz J, Hernandez AF, O'Connor CM, et al. Effect of Vericiguat vs Placebo on Quality of Life in Patients With Heart Failure and Preserved Ejection Fraction: The VITALITY-HFpEF Randomized Clinical Trial. JAMA. 2020;324(15):1512-1521. - Mentz RJ, Xu H, O'Brien EC, Thomas L, Alexy T, Gupta B, et al. PROVIDE-HF primary results: Patient-Reported Outcomes inVestigation following Initiation of Drug therapy with Entresto (sacubitril/valsartan) in heart failure. Am Heart J. 2020;230:35-43. - Bilgen F, Chen P, Poggi A, Wells J, Trumble E, Helmke S, et al. Insufficient Calorie Intake Worsens Post-Discharge Quality of Life and Increases Readmission Burden in Heart Failure. JACC Heart Fail. 2020;8(9):756-764. - Angermann CE, Assmus B, Anker SD, Asselbergs FW, Brachmann J, Brett ME, et al. Pulmonary artery pressure-guided therapy in ambulatory patients with symptomatic heart failure: the CardioMEMS European Monitoring Study for Heart Failure (MEMS-HF). Eur J Heart Fail. 2020;22(10):1891-1901. - Tummalapalli SL, Zelnick LR, Andersen AH, Christenson RH, deFilippi CR, Deo R, et al. Association of Cardiac Biomarkers With the Kansas City Cardiomyopathy Questionnaire in Patients With Chronic Kidney Disease Without Heart Failure. J Am Heart Assoc. 2020;9(13):e014385. - Damluji AA, Rodriguez G, Noel T, Davis L, Dahya V, Tehrani B, et al. Sarcopenia and health-related quality of life in older adults after transcatheter aortic valve replacement. Am Heart J. 2020;224:171-181. - Kosiborod MN, Jhund PS, Docherty KF, Diez M, Petrie MC, Verma S, et al. Effects of Dapagliflozin on Symptoms, Function, and Quality of Life in Patients With Heart Failure and Reduced Ejection Fraction: Results From the DAPA-HF Trial. Circulation. 2020;141(2):90-99. - Chandra A, Vaduganathan M, Lewis EF, Claggett BL, Rizkala AR, Wang W, et al. Health-Related Quality of Life in Heart Failure With Preserved Ejection Fraction: The PARAGON-HF Trial. JACC Heart Fail. 2019;7(10):862-874. - Denfeld QE, Lee CS, Woodward WR, Hiatt SO, Mudd JO, Habecker BA. Sympathetic Markers are Different Between Clinical Responders and Nonresponders After Left Ventricular Assist Device Implantation. J Cardiovasc Nurs. 2019;34(4):E1-E10. - Patel RB, Vaduganathan M, Felker GM, Butler J, Redfield MM, Shah SJ. Physical Activity, Quality of Life, and Biomarkers in Atrial Fibrillation and Heart Failure With Preserved Ejection Fraction (from the NEAT-HFpEF Trial). Am J Cardiol. 2019;123(10):1660-1666. - Dewan P, Rørth R, Jhund PS, Shen L, Raparelli V, Petrie MC, et al. Differential Impact of Heart Failure With Reduced Ejection Fraction on Men and Women. J Am Coll Cardiol. 2019;73(1):29-40. - Inohara T, Manandhar P, Kosinski AS, Matsouaka RA, Kohsaka S, Mentz RJ, et al. Association of Renin-Angiotensin Inhibitor Treatment With Mortality and Heart Failure Readmission in Patients With Transcatheter Aortic Valve Replacement. JAMA. 2018;320(21):2231-2241. - ​​Borlaug BA, Anstrom KJ, Lewis GD, Shah SJ, Levine JA, Koepp GA, et al. Effect of Inorganic Nitrite vs Placebo on Exercise Capacity Among Patients With Heart Failure With Preserved Ejection Fraction: The INDIE-HFpEF Randomized Clinical Trial. JAMA. 2018;320(17):1764-1773 - Tromp J, Tay WT, Ouwerkerk W, Teng TK, Yap J, MacDonald MR, et al. Multimorbidity in patients with heart failure from 11 Asian regions: A prospective cohort study using the ASIAN-HF registry. PLoS Med. 2018;15(3):e1002541. - Pressler A, Förschner L, Hummel J, Haller B, Christle JW, Halle M. Long-term effect of exercise training in patients after transcatheter aortic valve implantation: Follow-up of the SPORT:TAVI randomised pilot study. Eur J Prev Cardiol. 2018;25(8):794-801. - Yeo TJ, Yeo PSD, Hadi FA, Cushway T, Lee KY, Yin FF, et al. Single-dose intravenous iron in Southeast Asian heart failure patients: A pilot randomized placebo-controlled study (PRACTICE-ASIA-HF). ESC Heart Fail. 2018;5(2):344-353. - Filippatos G, Maggioni AP, Lam CSP, Pieske-Kraigher E, Butler J, Spertus J, et al. Patient-reported outcomes in the SOluble guanylate Cyclase stimulatoR in heArT failurE patientS with PRESERVED ejection fraction (SOCRATES-PRESERVED) study. Eur J Heart Fail. 2017;19(6):782-791. - Sherwood A, Blumenthal JA, Koch GG, Hoffman BM, Watkins LL, Smith PJ, et al. Effects of Coping Skills Training on Quality of Life, Disease Biomarkers, and Clinical Outcomes in Patients With Heart Failure: A Randomized Clinical Trial. Circ Heart Fail. 2017;10(1):e003410. - Zamani P, Tan V, Soto-Calderon H, Beraun M, Brandimarto JA, Trieu L, et al. Pharmacokinetics and Pharmacodynamics of Inorganic Nitrate in Heart Failure With Preserved Ejection Fraction. Circ Res. 2017;120(7):1151-1161. - Liu LC, Hummel YM, van der Meer P, Berger RM, Damman K, van Veldhuisen DJ, et al. Effects of sildenafil on cardiac structure and function, cardiopulmonary exercise testing and health-related quality of life measures in heart failure patients with preserved ejection fraction and pulmonary hypertension. Eur J Heart Fail. 2017;19(1):116-125. - Maulik SK, Wilson V, Seth S, Bhargava B, Dua P, Ramakrishnan S, et al. Clinical efficacy of water extract of stem bark of Terminalia arjuna (Roxb. ex DC.) Wight & Arn. in patients of chronic heart failure: a double-blind, randomized controlled trial. Phytomedicine. 2016;23(11):1211-9. - Hamshere S, Arnous S, Choudhury T, Choudry F, Mozid A, Yeo C, et al. Randomized trial of combination cytokine and adult autologous bone marrow progenitor cell administration in patients with non-ischaemic dilated cardiomyopathy: the REGENERATE-DCM clinical trial. Eur Heart J. 2015;36(44):3061-9. - Givertz MM, Anstrom KJ, Redfield MM, Deswal A, Haddad H, Butler J, et al. Effects of Xanthine Oxidase Inhibition in Hyperuricemic Heart Failure Patients: The Xanthine Oxidase Inhibition for Hyperuricemic Heart Failure Patients (EXACT-HF) Study. Circulation. 2015;131(20):1763-71. - Cosmi F, Di Giulio P, Masson S, Finzi A, Marfisi RM, Cosmi D, et al. Regular wine consumption in chronic heart failure: impact on outcomes, quality of life, and circulating biomarkers. Circ Heart Fail. 2015;8(3):428-37. - Colin-Ramirez E, McAlister FA, Zheng Y, Sharma S, Armstrong PW, Ezekowitz JA. The long-term effects of dietary sodium restriction on clinical outcomes in patients with heart failure. The SODIUM-HF (Study of Dietary Intervention Under 100 mmol in Heart Failure): a pilot study. Am Heart J. 2015;169(2):274-281.e1. - Abraham WT, Aggarwal S, Prabhu SD, Cecere R, Pamboukian SV, Bank AJ, et al. Ambulatory extra-aortic counterpulsation in patients with moderate to severe chronic heart failure. JACC Heart Fail. 2014;2(5):526-33. - Piña IL, Lin L, Weinfurt KP, Isitt JJ, Whellan DJ, Schulman KA, et al. Hemoglobin, exercise training, and health status in patients with chronic heart failure (from the HF-ACTION randomized controlled trial). Am J Cardiol. 2013;112(7):971-6. - Filippatos G, Farmakis D, Colet JC, Dickstein K, Lüscher TF, Willenheimer R, et al. Intravenous ferric carboxymaltose in iron-deficient chronic heart failure patients with and without anaemia: a subanalysis of the FAIR-HF trial. Eur J Heart Fail. 2013;15(11):1267-76. - Maurer MS, Teruya S, Chakraborty B, Helmke S, Mancini D. Treating anemia in older adults with heart failure with a preserved ejection fraction with epoetin alfa: single-blind randomized clinical trial of safety and efficacy. Circ Heart Fail. 2013;6(2):254-63. - Costanzo MR, Ivanhoe RJ, Kao A, Anand IS, Bank A, Boehmer J, et al. Prospective evaluation of elastic restraint to lessen the effects of heart failure (PEERLESS-HF) trial. J Card Fail. 2012;18(6):446-58. - Costanzo MR, Heywood JT, Massie BM, Iwashita J, Henderson L, Mamatsashvili M, et al. A double-blind, randomized, parallel, placebo-controlled study examining the effect of cross-linked polyelectrolyte in heart failure patients with chronic kidney disease. Eur J Heart Fail. 2012;14(8):922-30. - Huff CM, Turer AT, Wang A. Correlations between physician-perceived functional status, patient-perceived health status, and cardiopulmonary exercise results in hypertrophic cardiomyopathy. Qual Life Res. 2013;22(3):647-52. - Comin-Colet J, Lainscak M, Dickstein K, Filippatos GS, Johnson P, Lüscher TF, et al. The effect of intravenous ferric carboxymaltose on health-related quality of life in patients with chronic heart failure and iron deficiency: a subanalysis of the FAIR-HF study. Eur Heart J. 2013;34(1):30-8. - Allen LA, Gheorghiade M, Reid KJ, Dunlay SM, Chan PS, Hauptman PJ, et al. Identifying patients hospitalized with heart failure at risk for unfavorable future quality of life. Circ Cardiovasc Qual Outcomes. 2011;4(4):389-98. - Adams KF Jr, Piña IL, Ghali JK, Wagoner LE, Dunlap SH, Schwartz TA, et al. Prospective evaluation of the association between hemoglobin concentration and quality of life in patients with heart failure. Am Heart J. 2009;158(6):965-71. - Athanasopoulos LV, Dritsas A, Doll HA, Cokkinos DV. Comparative value of NYHA functional class and quality-of-life questionnaire scores in assessing heart failure. J Cardiopulm Rehabil Prev. 2010;30(2):101-5. - Flynn KE, Lin L, Ellis SJ, Russell SD, Spertus JA, Whellan DJ, et al. Outcomes, health policy, and managed care: relationships between patient-reported outcome measures and clinical measures in outpatients with heart failure. Am Heart J. 2009;158(4 Suppl):S64-71. - Gottlieb SS, Kop WJ, Ellis SJ, Binkley P, Howlett J, O'Connor C, et al. Relation of depression to severity of illness in heart failure (from Heart Failure And a Controlled Trial Investigating Outcomes of Exercise Training [HF-ACTION]). Am J Cardiol. 2009;103(9):1285-9. - Karavidas A, Parissis J, Arapi S, Farmakis D, Korres D, Nikolaou M, et al. Effects of functional electrical stimulation on quality of life and emotional stress in patients with chronic heart failure secondary to ischaemic or idiopathic dilated cardiomyopathy: a randomised, placebo-controlled trial. Eur J Heart Fail. 2008;10(7):709-13. - van Veldhuisen DJ, Dickstein K, Cohen-Solal A, Lok DJ, Wasserman SM, Baker N, et al. Randomized, double-blind, placebo-controlled study to evaluate the effect of two dosing regimens of darbepoetin alfa in patients with heart failure and anaemia. Eur Heart J. 2007;28(18):2208-16. - Parissis JT, Papadopoulos C, Nikolaou M, Bistola V, Farmakis D, Paraskevaidis I, et al. Effects of levosimendan on quality of life and emotional stress in advanced heart failure patients. Cardiovasc Drugs Ther. 2007;21(4):263-8. - Konstam MA, Gheorghiade M, Burnett JC Jr, Grinfeld L, Maggioni AP, Swedberg K, et al. Effects of oral tolvaptan in patients hospitalized for worsening heart failure: the EVEREST Outcome Trial. JAMA. 2007;297(12):1319-31. - Ponikowski P, Anker SD, Szachniewicz J, Okonko D, Ledwidge M, Zymlinski R, et al. Effect of darbepoetin alfa on exercise tolerance in anemic patients with symptomatic chronic heart failure: a randomized, double-blind, placebo-controlled trial. J Am Coll Cardiol. 2007;49(7):753-62. - Myers J, Zaheer N, Quaglietti S, Madhavan R, Froelicher V, Heidenreich P. Association of functional and health status measures in heart failure. J Card Fail. 2006;12(6):439-45 - Luther SA, McCullough PA, Havranek EP, Rumsfeld JS, Jones PG, Heidenreich PA, et al. The relationship between B-type natriuretic peptide and health status in patients with heart failure. J Card Fail. 2005;11(6):414-21. |
| Leicester Cough Questionnaire (LCQ) | - Bartley J, Garrett J, Camargo CA Jr, Scragg R, Vandal A, Sisk R, et al. Vitamin D(3) supplementation in adults with bronchiectasis: A pilot study. Chron Respir Dis. 2018;15(4):384-392. |
| Living with Neurologically Based Speech Difficulties (Living with Dysarthria, LwD) | - Lirani-Silva C, Mourão LF, Gobbi LT. Dysarthria and Quality of Life in neurologically healthy elderly and patients with Parkinson's disease. Codas. 2015;27(3):248-54. |
| Low Luminance Questionnaire (LLQ) | - Green J, Tolley C, Bentley S, Arbuckle R, Burstedt M, Whelan J, et al. Qualitative Interviews to Better Understand the Patient Experience and Evaluate Patient-Reported Outcomes (PRO) in RLBP1 Retinitis Pigmentosa (RLBP1 RP). Adv Ther. 2020;37(6):2884-2901. |
| Mini-Osteoporosis Quality of Life Questionnaire (Mini-OQLQ) | - Salaffi F, Cimmino MA, Malavolta N, Carotti M, Di Matteo L, Scendoni P, et al. The burden of prevalent fractures on health-related quality of life in postmenopausal women with osteoporosis: the IMOF study. J Rheumatol. 2007;34(7):1551-60. |
| Minnesota Living With Heart Failure Questionnaire (MLHFQ) | - Florea V, Rieger AC, Natsumeda M, Tompkins BA, Banerjee MN, Schulman IH, et al. The impact of patient sex on the response to intramyocardial mesenchymal stem cell administration in patients with non-ischaemic dilated cardiomyopathy. Cardiovasc Res. 2020;116(13):2131-2141. - Melo DTP, Nerbass FB, Sayegh ALC, Souza FR, Hotta VT, Salemi VMC, et al. Impact of pericardiectomy on exercise capacity and sleep of patients with chronic constrictive pericarditis. PLoS One. 2019;14(10):e0223838. - Coats CJ, Pavlou M, Watkinson OT, Protonotarios A, Moss L, Hyland R, et al. Effect of Trimetazidine Dihydrochloride Therapy on Exercise Capacity in Patients With Nonobstructive Hypertrophic Cardiomyopathy: A Randomized Clinical Trial. JAMA Cardiol. 2019;4(3):230-235. - Weinmann K, Werner J, Koenig W, Rottbauer W, Walcher D, Keßler M. Add-on Immunoadsorption Shortly-after Optimal Medical Treatment Further Significantly and Persistently Improves Cardiac Function and Symptoms in Recent-Onset Heart Failure-A Single Center Experience. Biomolecules. 2018;8(4):133. - Halbach M, Abraham WT, Butter C, Ducharme A, Klug D, Little WC, et al. Baroreflex activation therapy for the treatment of heart failure with reduced ejection fraction in patients with and without coronary artery disease. Int J Cardiol. 2018;266:187-192. - Florea V, Rieger AC, DiFede DL, El-Khorazaty J, Natsumeda M, Banerjee MN, et al. Dose Comparison Study of Allogeneic Mesenchymal Stem Cells in Patients With Ischemic Cardiomyopathy (The TRIDENT Study). Circ Res. 2017;121(11):1279-1290. - Ohlow MA, Brunelli M, Schreiber M, Lauer B. Therapeutic effect of immunoadsorption and subsequent immunoglobulin substitution in patients with dilated cardiomyopathy: Results from the observational prospective Bad Berka Registry. J Cardiol. 2017;69(2):409-416. - Arturi F, Succurro E, Miceli S, Cloro C, Ruffo M, Maio R, et al. Liraglutide improves cardiac function in patients with type 2 diabetes and chronic heart failure. Endocrine. 2017;57(3):464-473. - Kuschyk J, Roeger S, Schneider R, Streitner F, Stach K, Rudic B, et al. Efficacy and survival in patients with cardiac contractility modulation: long-term single center experience in 81 patients. - Abraham WT, Aggarwal S, Prabhu SD, Cecere R, Pamboukian SV, Bank AJ, et al. Ambulatory extra-aortic counterpulsation in patients with moderate to severe chronic heart failure. JACC Heart Fail. 2014;2(5):526-33. - Abdel-Salam Z, Rayan M, Saleh A, Abdel-Barr MG, Hussain M, Nammas W. I(f) current inhibitor ivabradine in patients with idiopathic dilated cardiomyopathy: Impact on the exercise tolerance and quality of life. Cardiol J. 2015;22(2):227-32. - Giusti II, Rodrigues CG, Salles FB, Sant'Anna RT, Eibel B, Han SW, et al. High doses of vascular endothelial growth factor 165 safely, but transiently, improve myocardial perfusion in no-option ischemic disease. Hum Gene Ther Methods. 2013;24(5):298-306. - Penn MS, Mendelsohn FO, Schaer GL, Sherman W, Farr M, Pastore J, et al. An open-label dose escalation study to evaluate the safety of administration of nonviral stromal cell-derived factor-1 plasmid to treat symptomatic ischemic heart failure. Circ Res. 2013;112(5):816-25. - Maurer MS, Teruya S, Chakraborty B, Helmke S, Mancini D. Treating anemia in older adults with heart failure with a preserved ejection fraction with epoetin alfa: single-blind randomized clinical trial of safety and efficacy. Circ Heart Fail. 2013;6(2):254-63. - Costanzo MR, Ivanhoe RJ, Kao A, Anand IS, Bank A, Boehmer J, et al. Prospective evaluation of elastic restraint to lessen the effects of heart failure (PEERLESS-HF) trial. J Card Fail. 2012;18(6):446-58. - Adams KF Jr, Piña IL, Ghali JK, Wagoner LE, Dunlap SH, Schwartz TA, et al. Prospective evaluation of the association between hemoglobin concentration and quality of life in patients with heart failure. Am Heart J. 2009;158(6):965-71. - Athanasopoulos LV, Dritsas A, Doll HA, Cokkinos DV. Comparative value of NYHA functional class and quality-of-life questionnaire scores in assessing heart failure. J Cardiopulm Rehabil Prev. 2010;30(2):101-5. - van Veldhuisen DJ, Dickstein K, Cohen-Solal A, Lok DJ, Wasserman SM, Baker N, et al. Randomized, double-blind, placebo-controlled study to evaluate the effect of two dosing regimens of darbepoetin alfa in patients with heart failure and anaemia. Eur Heart J. 2007;28(18):2208-16. - Birks EJ, Tansley PD, Hardy J, George RS, Bowles CT, Burke M, et al. Left ventricular assist device and drug therapy for the reversal of heart failure. N Engl J Med. 2006;355(18):1873-84. - Wojnicz R, Nowak J, Szyguła-Jurkiewicz B, Wilczek K, Lekston A, Trzeciak P, et al. Adjunctive therapy with low-molecular-weight heparin in patients with chronic heart failure secondary to dilated cardiomyopathy: one-year follow-up results of the randomized trial. Am Heart J. 2006;152(4):713.e1-7. - Keteyian SJ, Brawner CA, Schairer JR, Levine TB, Levine AB, Rogers FJ, et al. Effects of exercise training on chronotropic incompetence in patients with heart failure. Am Heart J. 1999;138(2 Pt 1):233-40. |
| Multiple System Atrophy Quality of Life questionnaire (MSA-QoL) | - Matsushima M, Yabe I, Oba K, Sakushima K, Mito Y, Takei A, et al. Comparison of Different Symptom Assessment Scales for Multiple System Atrophy. Cerebellum. 2016;15(2):190-200. |
| National Eye Institute Refractive Error Quality of Life Instrument - 42 (NEI RQL-42) | - Ren Q, Yang B, Liu L, Cho P. Orthokeratology in adults and factors affecting success: Study design and preliminary results. Cont Lens Anterior Eye. 2020;43(6):595-601. - González-Pérez J, Sánchez García Á, Villa-Collar C. Vision-Specific Quality of Life: Laser-Assisted in situ Keratomileusis Versus Overnight Contact Lens Wear. Eye Contact Lens. 2019;45(1):34-39 - Hays RD, Tarver ME, Spritzer KL, Reise S, Hilmantel G, Hofmeister EM, et al. Assessment of the Psychometric Properties of a Questionnaire Assessing Patient-Reported Outcomes With Laser In Situ Keratomileusis (PROWL). JAMA Ophthalmol. 2017;135(1):3-12. - Lipson MJ, Sugar A, Musch DC. Overnight corneal reshaping versus soft disposable contact lenses: vision-related quality-of-life differences from a randomized clinical trial. Optom Vis Sci. 2005;82(10):886-91. - Lipson MJ, Sugar A, Musch DC. Overnight corneal reshaping versus soft daily wear: a visual quality of life study (interim results). Eye Contact Lens. 2004;30(4):214-7. - Schmidt GW, Yoon M, McGwin G, Lee PP, McLeod SD. Evaluation of the relationship between ablation diameter, pupil size, and visual function with vision-specific quality-of-life measures after laser in situ keratomileusis. Arch Ophthalmol. 2007;125(8):1037-42. - Nichols JJ, Twa MD, Mitchell GL. Sensitivity of the National Eye Institute Refractive Error Quality of Life instrument to refractive surgery outcomes. J Cataract Refract Surg. 2005;31(12):2313-8. |
| National Eye Institute Visual Functioning Questionnaire - 25 (NEI VFQ-25) | - Green J, Tolley C, Bentley S, Arbuckle R, Burstedt M, Whelan J, et al. Qualitative Interviews to Better Understand the Patient Experience and Evaluate Patient-Reported Outcomes (PRO) in RLBP1 Retinitis Pigmentosa (RLBP1 RP). Adv Ther. 2020;37(6):2884-2901. - Afsharian P, Nolan-Kenney R, Lynch AE, Balcer LJ, Lynch DR. Correlation of Visual Quality of Life With Clinical and Visual Status in Friedreich Ataxia. J Neuroophthalmol. 2020;40(2):213-217. - Ihl T, Kadas EM, Oberwahrenbrock T, Endres M, Klockgether T, Schroeter J, et al. Investigation of Visual System Involvement in Spinocerebellar Ataxia Type 14. Cerebellum. 2020;19(4):469-482. - Hays RD, Tarver ME, Spritzer KL, Reise S, Hilmantel G, Hofmeister EM, et al. Assessment of the Psychometric Properties of a Questionnaire Assessing Patient-Reported Outcomes With Laser In Situ Keratomileusis (PROWL). JAMA Ophthalmol. 2017;135(1):3-12. - McLean RJ, Maconachie GD, Gottlob I, Maltby J. The Development of a Nystagmus-Specific Quality-of-Life Questionnaire. Ophthalmology. 2016;123(9):2023-7. - Khanna CL, Leske DA, Holmes JM. Factors Associated With Health-Related Quality of Life in Medically and Surgically Treated Patients With Glaucoma. JAMA Ophthalmol. 2018;136(4):348-355. - Kedar S, Ghate D, Murray EL, Corbett JJ, Subramony SH. Vision related quality of life in spinocerebellar ataxia. J Neurol Sci. 2015;358(1-2):404-8. - Tandon AK, Velez FG, Isenberg SJ, Demer JL, Pineles SL. Binocular inhibition in strabismic patients is associated with diminished quality of life. J AAPOS. 2014;18(5):423-6. - Hatt SR, Leske DA, Holmes JM. Responsiveness of health-related quality-of-life questionnaires in adults undergoing Strabismus surgery. Ophthalmology. 2010;117(12):2322-2328.e1. - Leske DA, Hatt SR, Holmes JM. Test-retest reliability of health-related quality-of-life questionnaires in adults with strabismus. Am J Ophthalmol. 2010;149(4):672-6. - Hatt SR, Leske DA, Bradley EA, Cole SR, Holmes JM. Comparison of quality-of-life instruments in adults with strabismus. Am J Ophthalmol. 2009;148(4):558-62 |
| Nystagmus-specific QOL questionnaire (NYS-29) | - McLean RJ, Maconachie GD, Gottlob I, Maltby J. The Development of a Nystagmus-Specific Quality-of-Life Questionnaire. Ophthalmology. 2016;123(9):2023-7. - Lingua RW, Gore C. Myectomy of the four horizontal rectus muscles with pulley fixation for the treatment of horizontal nystagmus in 10 adults: a pilot study. J AAPOS. 2020;24(2):80.e1-80.e6. |
| Osteoporosis Assessment Questionnaire (OPAQ) | - Silverman SL, Piziak VK, Chen P, Misurski DA, Wagman RB. Relationship of health related quality of life to prevalent and new or worsening back pain in postmenopausal women with osteoporosis. J Rheumatol. 2005;32(12):2405-9. |
| Osteoporosis Quality of Life Questionnaire (OQLQ) | - Measuring quality of life in women with osteoporosis. Osteoporosis Quality of Life Study Group. Osteoporos Int. 1997;7(5):478-87. |
| Osteoporosis-Targeted Quality of Life (OPTQoL) | - Chandler JM, Martin AR, Girman C, Ross PD, Love-McClung B, Lydick E, et al. Reliability of an Osteoporosis-Targeted Quality of Life Survey Instrument for use in the community: OPTQoL. Osteoporos Int. 1998;8(2):127-35. |
| Pediatric Eye Questionnaire (PedEyeQ) | - Birch EE, Castañeda YS, Cheng-Patel CS, Morale SE, Kelly KR, Jost RM, et al. Associations of Eye-Related Quality of Life With Vision, Visuomotor Function, and Self-Perception in Children With Strabismus and Anisometropia. Invest Ophthalmol Vis Sci. 2020;61(11):22. |
| Pittsburgh Insomnia Rating Scale (PIRS) | - Nasiri Lari Z, Hajimonfarednejad M, Riasatian M, Abolhassanzadeh Z, Iraji A, Vojoud M, et al. Efficacy of inhaled Lavandula angustifolia Mill. Essential oil on sleep quality, quality of life and metabolic control in patients with diabetes mellitus type II and insomnia. J Ethnopharmacol. 2020;251:112560. |
| Quality of Life Impact of Refractive Correction (QIRC) | - Zhao F, Zhao G, Zhao Z. Investigation of the Effect of Orthokeratology Lenses on Quality of Life and Behaviors of Children. Eye Contact Lens. 2018;44(5):335-338. |
| Quality of Life in Childhood Epilepsy Questionnaire (QOLCE-55) | - Sajobi TT, Wang M, Ferro MA, Brobbey A, Goodwin S, Speechley KN, et al. Multivariate trajectories across multiple domains of health-related quality of life in children with new-onset epilepsy. Epilepsy Behav. 2017;75:72-78. |
| Quality of Life in Childhood Epilepsy Questionnaire (QOLCE) | - Krueger DA, Care MM, Holland K, Agricola K, Tudor C, Mangeshkar P, et al. Everolimus for subependymal giant-cell astrocytomas in tuberous sclerosis. N Engl J Med. 2010;363(19):1801-11. |
| Quality Of Life In Epilepsy (QOLIE-10) | - Sarangi SC, Kaur N, Tripathi M. Assessment of psychiatric and behavioral adverse effects of antiepileptic drugs monotherapy: Could they have a neuroendocrine correlation in persons with epilepsy?. Epilepsy Behav. 2019;100(Pt A):106439. - Beran R, Berkovic S, Black A, Danta G, Dunne J, Frasca J, et al. AUStralian study of titration to effect profile of safety (AUS-STEPS): high-dose gabapentin (neurontin) in partial seizures. Epilepsia. 2001;42(10):1335-9. |
| QOLIE-10-P | - Steinhoff BJ, Wendling AS. Short-term impact of the switch from immediate-release to extended-release oxcarbazepine in epilepsy patients on high dosages. Epilepsy Res. 2009;87(2-3):256-9. |
| QOLIE-31 | - Ruggles KH, Haessly SM, Berg RL. Prospective study of seizures in the elderly in the Marshfield Epidemiologic Study Area (MESA). Epilepsia. 2001;42(12):1594-9. - Kim SH, Lim SC, Kim W, Kwon OH, Kim CM, Lee JM, et al. Changes in background electroencephalography and regional cerebral glucose metabolism in focal epilepsy patients after 1-month administration of levetiracetam. Neuropsychiatr Dis Treat. 2015;11:215-23. - Richardson SP, Farias ST, Lima AR 3rd, Alsaadi TM. Improvement in seizure control and quality of life in medically refractory epilepsy patients converted from polypharmacy to monotherapy. Epilepsy Behav. 2004;5(3):343-7. |
| QOLIE for Adolescents (QOLIE-AD-48) | - Turky A, Beavis JM, Thapar AK, Kerr MP. Psychopathology in children and adolescents with epilepsy: an investigation of predictive variables. Epilepsy Behav. 2008;12(1):136-44. |
| Quality of Life in Essential Tremor Questionnaire (QUEST) | - Degeneffe A, Kuijf ML, Ackermans L, Temel Y, Kubben PL. Comparing deep brain stimulation in the ventral intermediate nucleus versus the posterior subthalamic area in essential tremor patients. Surg Neurol Int. 2018;9:244. - Mohammed N, Patra D, Nanda A. A meta-analysis of outcomes and complications of magnetic resonance-guided focused ultrasound in the treatment of essential tremor. Neurosurg Focus. 2018;44(2):E4. - Zaaroor M, Sinai A, Goldsher D, Eran A, Nassar M, Schlesinger I. Magnetic resonance-guided focused ultrasound thalamotomy for tremor: a report of 30 Parkinson's disease and essential tremor cases. J Neurosurg. 2018;128(1):202-210. |
| Quality of Life in the Dysarthric Speaker (QOL-Dys) | - Piacentini V, Mauri I, Cattaneo D, Gilardone M, Montesano A, Schindler A. Relationship between quality of life and dysarthria in patients with multiple sclerosis. Arch Phys Med Rehabil. 2014;95(11):2047-54. - Piacentini V, Zuin A, Cattaneo D, Schindler A. Reliability and validity of an instrument to measure quality of life in the dysarthric speaker. Folia Phoniatr Logop. 2011;63(6):289-95. |
| Quality of Life questionnaire In Osteoporosis (QUALIOST) | - Marquis P, Roux C, de la Loge C, Diaz-Curiel M, Cormier C, Isaia G, et al. Strontium ranelate prevents quality of life impairment in post-menopausal women with established vertebral osteoporosis. Osteoporos Int. 2008;19(4):503-10. |
| Quality-of-Life Scale for Myopia developed by Erikson et al. (2004) | - Erickson DB, Stapleton F, Erickson P, du Toit R, Giannakopoulos E, Holden B. Development and validation of a multidimensional quality-of-life scale for myopia. Optom Vis Sci. 2004;81(2):70-81. |
| Questionnaire for Evaluating Quality of Life of Pathologic Myopia Patients by Takashima T et al (2001) | - Takashima T, Yokoyama T, Futagami S, Ohno-Matsui K, Tanaka H, Tokoro T, et al. The quality of life in patients with pathologic myopia. Jpn J Ophthalmol. Jan-Feb 2001;45(1):84-92. |
| Questionnaire on the impact of strabismus on patient quality of life by de Barros Ribeiro G et al (2014) | - de Barros Ribeiro G, Bach AG, Faria CM, Anastásia S, Almeida HC. Quality of life of patients with strabismus. Arq Bras Oftalmol. 2014;77(2):110-3. |
| Scleroderma gastrointestinal tract 1.0 questionnaire (SSC-GIT 1.0) | - Yang H, Xu D, Li MT, Yao Y, Jin M, Zeng XF, et al. Gastrointestinal manifestations on impaired quality of life in systemic sclerosis. J Dig Dis. 2019;20(5):256-261. |
| Scoliosis Quality of Life Index (SQLI) | - Parent EC, Hill D, Moreau M, Mahood J, Raso J, Lou E. Score Distribution of the Scoliosis Quality of Life Index Questionnaire in Different Subgroups of Patients With Adolescent Idiopathic Scoliosis. Spine (Phila Pa 1976). 2007;32(16):1767-77. |
| Scoliosis Research Society 7-item (SRS-7) | - Jain A, Sponseller PD, Negrini S, Newton PO, Cahill PJ, Bastrom TP, et al. SRS-7: A Valid, Responsive, Linear, and Unidimensional Functional Outcome Measure for Operatively Treated Patients With AIS. Spine (Phila Pa 1976). 2015;40(9):650-5. |
| Scoliosis Research Society 30-item (SRS-30) | - Ibrahim JM, Singh P, Beckerman D, Hu SS, Tay [B](https://pubmed.ncbi.nlm.nih.gov/32206514/#affiliation-1), Deviren [V](https://pubmed.ncbi.nlm.nih.gov/32206514/#affiliation-1), Burch S, Berven SH, Outcomes and Quality of Life Improvement After Multilevel Spinal Fusion in Elderly Patients, Global Spine J. 2020 Apr;10(2):153-159 - Johnston CE, Tran DP, McClung A, Functional and Radiographic Outcomes Following Growth-Sparing Management of Early-Onset Scoliosis, J Bone Joint Surg Am. 2017 Jun 21;99(12):1036-1042. |
| Scoliosis Research Society 22-item (: SRS-22(r) | - Cheshire J, Gardner A, Berryman F, Pynsent P. Do the SRS-22 self-image and mental health domain scores reflect the degree of asymmetry of the back in adolescent idiopathic scoliosis?. Scoliosis Spinal Disord. 2017;12:37. - Carreon LY, Sanders JO, Diab M, Sucato DJ, Sturm PF, Glassman SD. The minimum clinically important difference in Scoliosis Research Society-22 Appearance, Activity, And Pain domains after surgical correction of adolescent idiopathic scoliosis. Spine (Phila Pa 1976). 2010;35(23):2079-83. |
| Short Form-Nepean Dyspepsia Index (SF-NDI) | - Dale HF, Jensen C, Hausken T, Valeur J, Hoff DAL, Lied GA. Effects of a Cod Protein Hydrolysate Supplement on Symptoms, Gut Integrity Markers and Fecal Fermentation in Patients with Irritable Bowel Syndrome. Nutrients. 2019;11(7):1635. |
| Short Osteoporosis Quality of Life Questionnaire (ECOS-16) | - Badia X, Díez-Pérez A, Lahoz R, Lizán L, Nogués X, Iborra J. The ECOS-16 questionnaire for the evaluation of health related quality of life in post-menopausal women with osteoporosis. Health Qual Life Outcomes. 2004;2:41. |
| Short Inflammatory Bowel Disease Questionnaire (SIBDQ) | - Pedersen N, Ankersen DV, Felding M, Wachmann H, Végh Z, Molzen L, et al. Low-FODMAP diet reduces irritable bowel symptoms in patients with inflammatory bowel disease. World J Gastroenterol. 2017;23(18):3356-3366. - Rampton DS, Goodhand JR, Joshi NM, Karim AB, Koodun Y, Barakat FM, et al. Oral Iron Treatment Response and Predictors in Anaemic Adolescents and Adults with IBD: A Prospective Controlled Open-Label Trial. J Crohns Colitis. 2017;11(6):706-715. - Castro FD, Magalhães J, Carvalho PB, Moreira MJ, Mota P, Cotter J. Lower Levels Of Vitamin D Correlate With Clinical Disease Activity And Quality Of Life In Inflammatory Bowel Disease. Arq Gastroenterol. 2015;52(4):260-5. - Koutroubakis IE, Ramos-Rivers C, Regueiro M, Koutroumpakis E, Click B, Schwartz M, et al. The Influence of Anti-tumor Necrosis Factor Agents on Hemoglobin Levels of Patients with Inflammatory Bowel Disease. Inflamm Bowel Dis. 2015;21(7):1587-93. - Hlavaty T, Krajcovicova A, Koller T, Toth J, Nevidanska M, Huorka M, et al. Higher vitamin D serum concentration increases health related quality of life in patients with inflammatory bowel diseases. World J Gastroenterol. 2014;20(42):15787-96. - Voiosu T, Benguş A, Dinu R, Voiosu AM, Bălănescu P, Băicuş C, et al. Rapid fecal calprotectin level assessment and the SIBDQ score can accurately detect active mucosal inflammation in IBD patients in clinical remission: a prospective study. J Gastrointestin Liver Dis. 2014;23(3):273-8. |
| Sino-Nasal Outcome Test (SNOT-20) | - Bartley J, Garrett J, Camargo CA Jr, Scragg R, Vandal A, Sisk R, et al. Vitamin D(3) supplementation in adults with bronchiectasis: A pilot study. Chron Respir Dis. 2018;15(4):384-392. - Marple B, Newcomer M, Schwade N, Mabry R. Natural history of allergic fungal rhinosinusitis: a 4- to 10-year follow-up. Otolaryngol Head Neck Surg. 2002;127(5):361-6. |
| Sino-Nasal Outcome Test (SNOT-22) | - Fu CH, Huang CC, Chen YW, Chang PH, Lee TJ. Nasal nitric oxide in relation to quality-of-life improvements after endoscopic sinus surgery. Am J Rhinol Allergy. 2015;29(6):e187-91. |
| Speech Handicap Index (SHI) | - Dwivedi RC, St Rose S, Roe JWG, Chisholm E, Elmiyeh B, Nutting CM, et al. First report on the reliability and validity of speech handicap index in native English-speaking patients with head and neck cancer. Head Neck. 201;33(3):341-8. |
| Swallowing Quality Of Life (SWAL-QOL) | - Keage MJ, Delatycki MB, Gupta I, Corben LA, Vogel AP. Dysphagia in Friedreich Ataxia. Dysphagia. 2017;32(5):626-635. |
| The Italian Spine Youth Quality of Life questionnaire (ISYQOL) | - Alanazi MH, Parent EC, Bettany-Saltikov J, Hill D, Southon S. Convergent validity, ceiling, and floor effects of the English-ISYQOL against established quality of life questionnaires (SRS-22r and SAQ) and curve angles in adolescents with idiopathic scoliosis. Stud Health Technol Inform. 2021;280:225-230. |
| University of California Los Angeles Prostate Cancer Index (UCLA-PCI) | - Hashine K, Kusuhara Y, Miura N, Shirato A, Sumiyoshi Y, Kataoka M. A prospective longitudinal study comparing a radical retropubic prostatectomy and permanent prostate brachytherapy regarding the health-related quality of life for localized prostate cancer. Jpn J Clin Oncol. 2008;38(7):480-5. |
| University of Washington Quality of Life (UWQOL) | - Thomas L, Jones TM, Tandon S, Carding P, Lowe D, Rogers S. Speech and voice outcomes in oropharyngeal cancer and evaluation of the University of Washington Quality of Life speech domain. Clin Otolaryngol. 2009;34(1):34-42. - Radford K, Woods H, Lowe D, Rogers SN. A UK multi-centre pilot study of speech and swallowing outcomes following head and neck cancer. Clin Otolaryngol Allied Sci. 2004;29(4):376-81. - Sadiq Z, Sammut S, Lopes V. Non-complex reconstructive techniques in the management of BRONJ: a case series of patient-related outcomes. Oral Maxillofac Surg. 2014;18(2):223-7. |
| Vision Quality of Life Questionnaire by McKeon C et al,. (1997) | - McKeon C, Wick B, Aday LA, Begley C. A case-comparison of intermittent exotropia and quality of life measurements. Optom Vis Sci. 1997;74(2):105-10. |
| Visual Function Scale-Plus (VFS-plus) | - Costela FM, Pesudovs K, Sandberg MA, Weigel-DiFranco C, Woods RL. Validation of a vision-related activity scale for patients with retinitis pigmentosa. Health Qual Life Outcomes. 2020;18(1):196. |
